# Spiked Systems for Colonic Drug Delivery: Architectural Opportunities and Quality Assurance of Selective Laser Sintering

**DOI:** 10.1021/acsbiomaterials.4c02038

**Published:** 2025-02-06

**Authors:** Angelos Gkaragkounis, Konstantina Chachlioutaki, Orestis L. Katsamenis, Fernando Alvarez-Borges, Savvas Koltsakidis, Ioannis Partheniadis, Nikolaos Bouropoulos, Ioannis S. Vizirianakis, Dimitrios Tzetzis, Ioannis Nikolakakis, Chris H. J. Verhoeven, Dimitrios G. Fatouros, Kjeld J. C. van Bommel

**Affiliations:** † Laboratory of Pharmaceutical Technology, Department of Pharmacy, School of Health Sciences, Aristotle University of Thessaloniki, Thessaloniki GR 54124, Greece; ‡ The Netherlands Organization for Applied Scientific Research (TNO), Eindhoven 5656 AE, The Netherlands; § Center for Interdisciplinary Research and Innovation (CIRI-AUTH), Thessaloniki 54124, Greece; ∥ μ-VIS X-Ray Imaging Centre, Faculty of Engineering and Physical Sciences, University of Southampton, Southampton SO17 1BJ, U.K.; ⊥ Institute for Life Sciences, University of Southampton, University Road, Highfield, Southampton SO17 1BJ, U.K.; # Digital Manufacturing and Materials Characterization Laboratory, School of Science and Technology, International Hellenic University, Thessaloniki 57001, Greece; ∇ Department of Materials Science, University of Patras, Patras 26504, Rio, Greece; ○ Institute of Chemical Engineering and High Temperature Chemical Processes, Foundation for Research and Technology Hellas, Patras 26504, Greece; ◆ Laboratory of Pharmacology, Department of Pharmacy, Aristotle University of Thessaloniki, Thessaloniki GR 54124, Greece

**Keywords:** selective laser sintering, 3D printing, spiked drug delivery systems, colonic drug delivery, mucoadhesion, loperamide, extended retention time

## Abstract

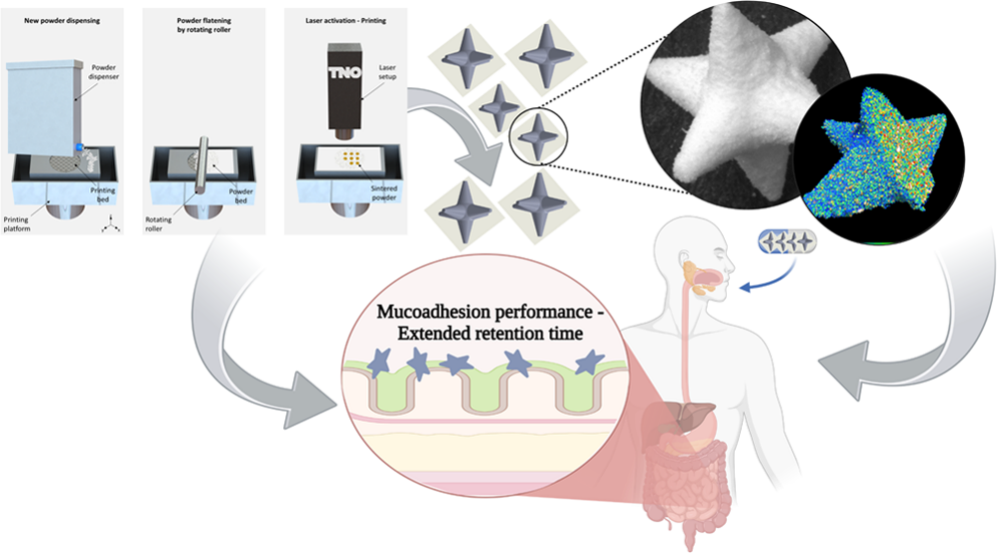

Additive manufacturing has been a breakthrough therapy for the pharmaceutical industry raising opportunities for long-quested properties, such as controlled drug-delivery. The aim of this study was to explore the geometrical capabilities of selective laser sintering (SLS) by creating spiked (tapered-edged) drug-loaded specimens for administration in colon. Poly(vinyl alcohol) (PVA) was used as the binding material and loperamide hydrochloride was incorporated as the active ingredient. Printing was feasible without the addition of a sintering agent or other additives. Innovative printing protocols were developed to help improve the quality of the obtained products. Intentional vibrations were applied on the powder bed through rapid movements of the printing platform in order to facilitate rigidity and consistency of the printed objects. The drug-loaded products had physicochemical properties that met the pharmacopoeia standards and exhibited good biocompatibility. The behavior of spiked balls (spherical objects with prominent spikes) and their retention time in the colon was assessed using a custom *ex vivo* intestinal setup. The spiked balls showed favorable mucoadhesive properties over the unspiked ones. No movement on the tissue was recorded for the spiked balls, and specimens with more spikes exhibited longer retention times and potentially, enhanced bioavailability. Our results suggest that SLS 3D printing is a versatile technology that holds the potential to revolutionize drug delivery systems by enabling the creation of complex geometries and medications with tunable properties.

## Introduction

Additive Manufacturing (AM) is the quintessence of customization. The shape freedom that AM offers is essential for the fabrication of the, often highly complex, architectures that are required for the realization of unusual properties and advanced functionalities. Despite the fact that the ball of AM (also known as “rapid prototyping” and “3D printing”) has been rolling in the pharmaceutical field since 1996, it is only recently that research has turned its focus on valuable materials and atypical geometries, in the attempt of creating drug-loaded products with unusual features and hard-to-reach therapeutic objectives.^1−4^

AM holds the promise of digitalizing the drug industry while enabling decentralized manufacturing at point-of-care (PoC) printing hubs.^5−8^ A recent study demonstrated, for the first time, the bioequivalence of 3D printed sildenafil tablets to their large-scale-produced marketed originator. After administration to twelve (12) healthy volunteers, the results are laying the groundwork for at PoC production of 3D printed medicines and testing in clinical trials with human participants.^9^ Well-established quality assurance (QA) and quality control (QC) protocols are pivotal for the realization of such a fundamental shift toward small-batch production on demand at PoC. To that end, recent research studies investigate mathematical modeling (e.g., Artificial Intelligence) for the prediction, and nondestructive analytical methods, for the evaluation of printed products’ properties.^10−16^ In a recent study, a data set of 170 different formulations was used for the development of Machine Learning models that predict the printability of pharmaceutical formulations by means of the selective laser sintering (SLS) process. The inputs included data retrieved from Fourier-transform infrared spectroscopy (FTIR), X-ray powder diffraction (XRPD) and Differential Scanning Calorimetry (DSC).^17^ In another study, Fourier-Transform Near Infrared Spectroscopy (FTNIR) was evaluated as a nondestructive method, able to predict the density and drug release from tablets fabricated with SLS printing.^18^

**Figure 1:**
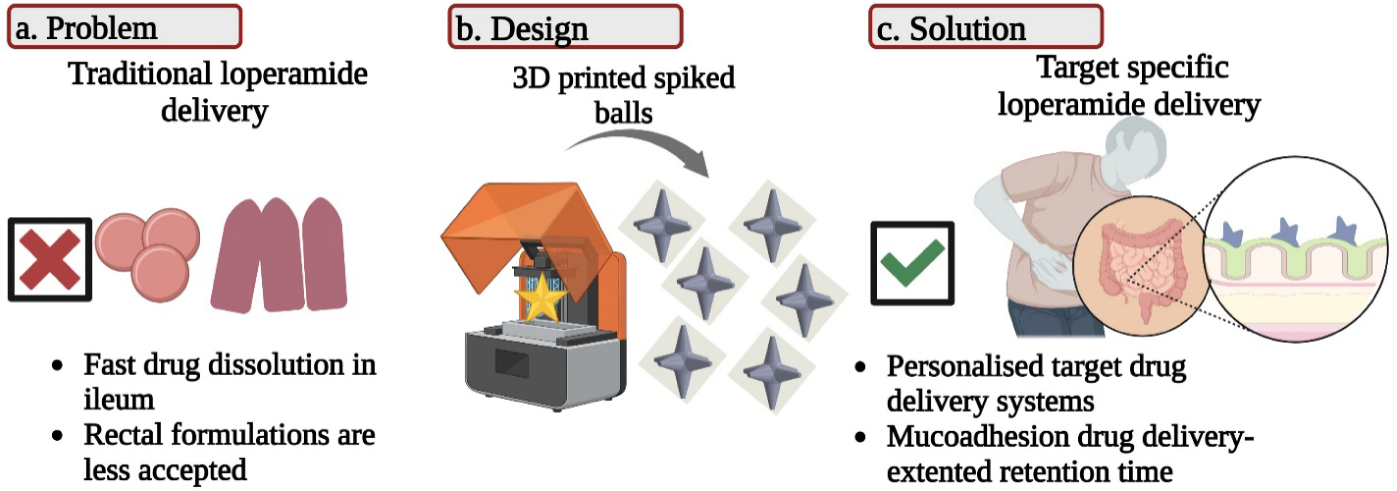
Conceptualization of the study: (a.) Traditional loperamide delivery results in a delayed onset of action because the drug dissolves in the ileum, which is not the intended target site. (b.) As a proof-of-concept, sharp-edged 3D-printed objects were designed using selective laser sintering. (c.) Mucoadhesive drug delivery systems were developed to increase retention time and adhesion to the target site. The spikes incorporated into the 3D-printed formulations enhance retention, extending the overall release of loperamide at the site of action. *Created with BioRender.com*.

Among numerous other advantages (e.g., tunable release profiles, personalized shape and size, multidrug combinations, preferable solid-state properties) SLS is a technique capable of producing relatively large batches of dosage forms due to the instrument’s usually large printing volume. SLS printing comprises exposure of fine powder particles to a narrow laser beam resulting in high resolution (down to hundreds of micrometers) and a high potential for fabricating complex architectures. As a powder bed technique, SLS has the unique characteristic that the printed structures remain immersed within and supported by the powder pool, allowing printing of extreme overhangs without additional supportive structures, as typically required during Fused Deposition Modeling (FDM) or Stereolithography (SLA) 3D printing.^11,16,18−22^

The oral route is the most common route of administration. However, drug absorption of orally administered dosage forms is being challenged by several obstacles, especially since the development of novel compounds that emerge from modern drug discovery (e.g., peptides, proteins etc.). Colon-targeted drug delivery enables a wide range of therapeutic benefits, with the most prominent ones being the direct access to local targets, reduction in systemic drug exposure and improvement of bioavailability.^23,24^ During the past decades, several devices have been proposed for addressing the challenges of oral delivery and targeting intestinal areas.^25^ With the traditional powder technology methods, particulate formulations are being compressed into tablets, with most of them comprising a central drug-loaded reservoir covered by protective polymeric materials that provide resistance in the harsh stomach environment and facilitate release upon intestinal triggers. However, due to their shape, these formulations are typically characterized by short residence times and poor contact with the intestinal wall. Lately, microfabricated drug-carrying polymeric devices have been proposed as promising systems for intestinal delivery, offering mucoadhesive properties and allowing unidirectional release of the active pharmaceutical ingredient (API).^26−29^ Recent studies demonstrate the *ex vivo* and *in vivo* potential of microcontainers, highlighting the influence of their geometry on retention time and bioavailability.^30−34^ Recently, a custom stereolithography 3D printer was used for the development of radiopaque microdevices with enhanced mucoadhesive geometries. After administration to rodents, the performance of three different designs was assessed. The results suggest that enhanced mucoadhesion might have occurred in some intestinal sites, but the overall retention time was not significantly increased. In addition, no preferred spatial orientation was observed, although the microcontainers had been designed in manners that would facilitate unidirectional distribution.^35,36^

The current study explores the fabrication capabilities of SLS 3D printing and its utilization for the development of drug-loaded dosage forms with mucoadhesive properties. Poly(vinyl alcohol) was used as a polymeric matrix and loperamide hydrochloride (LOP) was incorporated as the API. In this work, we designed and fabricated, for the first time, spiked formulations inspired by historical weapons using SLS. This approach highlights the challenges associated with this method,^37^ notably achieving successful fabrication without the use of a sintering agent or prior-printing heating of the powder bed. The materials and printed products were thoroughly studied for their physicochemical and drug-release characteristics. Upon oral administration via a gastro-resistant capsule, the spiked geometries were hypothesized to prolong retention time in the intestinal tract and enhance bioavailability, thereby demonstrating the second aspect of the study’s novelty. Their behavior and performance were further evaluated using an *ex vivo* intestinal model. This functional application underscores the potential of SLS 3D printing as a fabrication method for colonic drug delivery systems with controlled properties (Figure 1).

## Materials and Methods

### Materials

Poly(vinyl alcohol) 4-88 (PVA, Parteck MXP), poly(vinyl alcohol) 3-82 (Parteck MXP), hydroxypropyl methylcellulose (HPMC), polycaprolactone and Eudragit L100 were obtained from Merck KGaA (Darmstadt, Germany). Vivapharm HPMC E50, Vivapharm HPMC E6, Vivapharm PVA 05 and Vivapharm PVP/VA were obtained from JRS Pharma GmbH (Rosenberg, Germany). Vinylpyrrolidone-vinyl acetate Kollidon VA64 was obtained from BASF (Ludwigshafen, Germany). Microcrystalline cellulose Pharmacel 102 was obtained from DFE Pharma (Goch, Germany). Loperamide Hydrochloride (LOP) was obtained from Xi’an Faithful Biotech Co., Ltd. (Xi’an, China). All other chemicals were of analytical grade and used as obtained.

### SLS 3D Printing Preparation

The SLS technique requires powder preparation prior printing. Each powder was sieved using a 106 μm stainless steel sieve to remove particle agglomerates and large particles. For mixtures, the sieved powder materials where mixed using a Turbula mixer for 15 min at 100 rpm. For the drug-loaded samples, LOP (8.00 wt %) was sieved and mixed with the polymeric matrix, as before.

**Figure 2:**
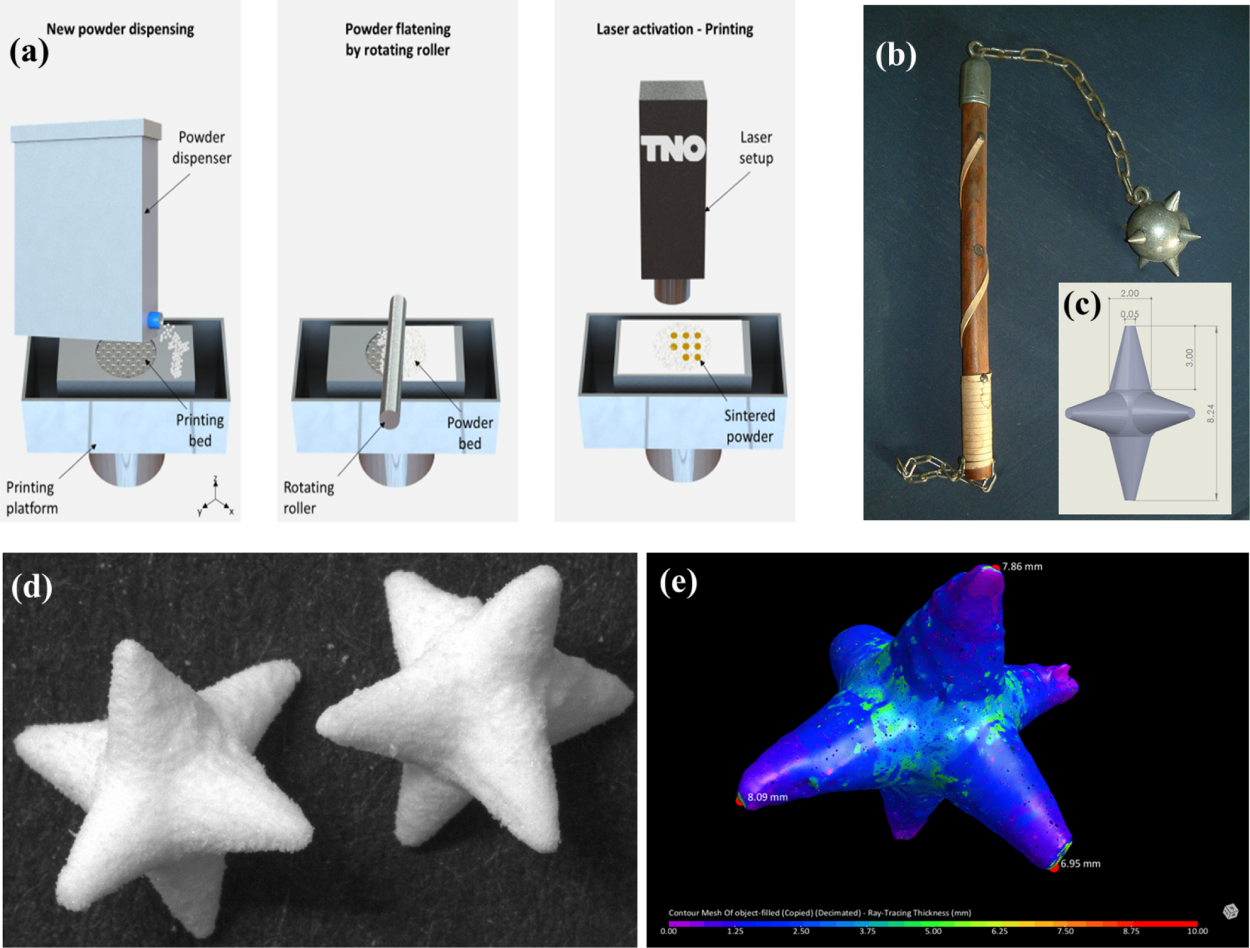
(a) Schematic representation of the components of the SLS setup which was used during this study. The printing platform moves on the X and Y axes, the printing bed moves on the Z axis and the rest of the parts are static. (b) A modern representation of a flail weapon,^48^ (c) CAD dimensions in mm, (d) 3D printed spiked balls, (e) Dimensional assessment using “ray-tracing thickness”. Color map [0.0 10.0] mm.

The 3D printer that was used in this study is a custom SLS setup developed by TNO (The Netherlands Organization for Applied Scientific Research, The Netherlands) (Figure 2a). It consists of a powder dispenser, a counter rotating roller that uniformly spreads the fresh powder and creates consistent layers, a moving platform (*X*, *Y* axes) with a circular printing bed (*r* = 50 mm, moving on *Z* axis) and a static CO_2_ laser setup (λ = 10.6 μm) which comprises a lens with a 0.143 mm wide focus point. The roller was cleaned, first with an isopropyl alcohol wet wipe and then with a dry wipe, before every printing job. The sieved powder or powder mixture was transferred to the powder dispenser. Repeated coating steps allowed for the initial coverage of the printing bed with the powder material.

The printing designs (tablets or spiked balls) were created in Solidworks 2020 (Dassault Systems, USA) and exported as.stl files. Subsequently, they were sliced with a modified preset of Simplify 3D Software 4.0.1, in order to be compatible with the SLS setup, and imported as.gcode files in the printing software, developed by TNO. The printing parameters were varied and depended on the thermal and optical properties of each material. Printing was carried out under ambient conditions (room temperature: 21.30 ± 0.73 °C, relative humidity: 45.51 ± 4.69%) with no additional heating in the printing environment.

### Geometries

Cylindrical tablets (9.00 mm in diameter, 4.00 mm in height) and spiked spheres with six edges were selected as test geometries for screening purposes (Figure 2d). The spiked balls consisted of a spherical core (diameter: 3.00 mm) with prominent tapered cylinders (base diameter: 2.00 mm, height: 3.00 mm, tip diameter: 0.15 mm) (Figure 2c). This design, inspired by the striking heads of medieval flail weapons (Figure 2b), has been hypothesized to result in increased retention time of the printed dosage forms in the gastrointestinal tract, upon release from a gastro-resistant capsule. Future studies will aim to further validate this hypothesis by optimizing the capsule dimensions and evaluating the design’s performance under *in vivo* conditions. The spikes can facilitate adhesion and potentially promote retention by increasing the contact area available for interaction with the epithelial surface and reducing the dislodging forces, as the spikes create resistance against the fluid flow.^29^

### Characterization of the 3D Printed Objects

#### Solid State Analysis

The X-ray diffractograms of the raw materials, unsintered powder blends and the 3D printed objects were recorded on a Scintag XDS 2000 diffractometer, using Cu Kα radiation within a 2θ range of 5° to 60°. The FTIR spectra were recorded on a Shimadzu IR Prestige-21 spectrophotometer (Shimadzu, Kyoto, Japan) at the wavenumber range of 4500–700 cm^–1^ and the DSC thermograms on a DSC apparatus (DSC 204 F1 Phoenix, Netzsch) in the temperature range between 25 and 250 °C under a nitrogen flow of 70 mL/min and a heating rate of 10 °C/min.

#### *In Vitro* Disintegration Studies

The disintegration study of the 3D printed balls was performed using the Petri dish method. Specifically, unspiked or spiked (2, 4, or 6 spikes) balls were placed in a Petri dish and subsequently 5 mL of water were added at room temperature. The disintegration process was recorded using a camera (Xiaomi Mi A2, Beijing, China, photo and video resolution: 12 megapixel and 1080 pixel).

#### *In Vitro* Dissolution Studies

The LOP content of the 3D printed tablets and spiked balls was quantified by HPLC analysis after dissolving the printed objects in a 2:1 mixture of methanol and distilled water. *In vitro* dissolution studies were performed in PBS pH 7.4 at 37 °C using the USP Type II dissolution apparatus (paddle) at 50 rpm. Samples (3 mL) were withdrawn at predetermined time intervals and the LOP content was quantified with HPLC analysis.

#### High Performance Liquid Chromatography (HPLC)

Loperamide quantification was performed in an HPLC system equipped with a pump (LC-10 AD VP), an autosampler (SIL-20A HT) with a 100 μL loop and a UV–vis detector model SPD-10A VP (Shimadzu, Kyoto, Japan). A Discovery HS C18 column (15 cm × 4.6 mm, 5 mm, Supelco, Sigma-Aldrich) was used for the analysis of loperamide, the mobile phase consisted of a mixture of 25 Mm phosphate buffer pH 2.8 and ACN (55:45% v/v) and the wavelength was set at 259 nm. Calibration curves were linear within the concentration range of 5–100 μg/mL in all tested media.

### In Vitro Assays in Eukaryotic Cells

#### Caco-2 Cell Experiments

Caco-2 cells, at passage 40, were cultured in DMEM with a pH of 7.4. The culture medium was supplemented with 10% fetal bovine serum, 1% nonessential amino acid solution, and 1% penicillin-streptomycin solution. These cells were cultured in a controlled environment at 37 °C with 5% CO_2_ and 95% air in a 90% relative humidity chamber. Before each experiment, the cells were rinsed with PBS and collected using trypsin. After introducing DMEM to halt trypsin activity, the cell culture was centrifuged at 1200 rpm for 5 min, and the cells were suspended in an appropriate volume of DMEM to achieve the desired cell density in each well.

#### In Vitro Cell Viability Assay

To evaluate the biocompatibility of LOP-loaded 3D printed products, a CCK-8 assay was conducted. For the cytotoxicity test, Caco-2 cells were seeded at a density of 3 × 10^4^ cells/cm^2^ in 96-well plates (100 μL per well) and allowed to attach over a 24-h period. These cells were exposed to different concentrations of pure LOP (varying concentration from 0.05 μg/mL to 10 μg/mL), LOP-loaded tablets in form of solutions (0.05 μg/mL to 10 μg/mL referred to LOP concentration in the tablet) and placebo tablets (in concentrations from 0.575 to 115 μg/mL, corresponding to the concentrations of the polymeric carriers in the loaded tablets). This exposure lasted for 24 h. Subsequently, the formulations were removed, cells were gently washed with PBS, and the cell viability test was performed. DMEM (100 μL per well) and Cell Counting Kit-8 (CCK8, St. Louis, MO, Sigma-Aldrich) reagent were added to each well. This mixture was then incubated for an additional 3 h at 37 °C. The optical density at 450 nm was measured using a multifunction microplate reader (Multiskan MS photometer type 352; Labsystems, Helsinki, Finland). Cell viability was calculated as a percentage based on the absorbance of each sample relative to the control.

#### Mucoadhesion Studies on Ex Vivo Intestinal Model

Fresh porcine large intestine was obtained from a local slaughterhouse and used within 24 h upon collection. The large intestine was carefully prepared by gently rinsing it with PBS solution (pH 7.4) to remove any residues. The tissue was then secured onto a flat surface and was cut open in a longitudinal direction. The mucoadhesive performance of unspiked balls and spiked balls with 0, 2, 4, or 6 spikes was assessed *ex vivo* on porcine mucosa from the ascending colon using a custom setup, specially designed for these experiments and similar to a previously reported setup.^31,38^ The instrumentation comprised a 3D printed tissue holder with adjustable angle and an environmental chamber (Figure S1a,b). A heating bath and a humidifier were used to maintain stability of temperature and humidity. The tissue holder was 19.50 cm in length and 3.30 cm in width. A peristaltic pump was used for the hydration of the intestinal tissue. More details regarding the experimental assembly can be found in (Supporting Information Section S1). Mucoadhesion studies were conducted in the designed setup. PBS was prepared at pH 7.4 and kept at 37 °C until further use. An 18 cm long piece of porcine large intestine was placed and secured on the tissue holder. Then, the tissue was placed at a 20*°* angle in the preheated (37 ± 1.1 °C) and with high-humidity (60.9 ± 11.5%) chamber.^39^ Next, the intestine was flushed with PBS for 15 min (4.1 mL/min) to remove all residues. After washing, three 3D printed objects of the same design were placed on the intestine, approximately 2 cm from the upper part of the tissue. Subsequently, the tissue holder was placed back at an angle of 20° and a flow of PBS 1.55 mL/min was initiated.^40^ The interaction was allowed to continue for 30 min and the entire procedure was recorded with a camera.

#### Defect Analysis and Porosity by Means of Microfocus Computed Tomography (μCT)

X-ray computed microtomography (μCT) was used to analyze the microstructure of the more complex printed objects (6-spiked balls), evaluating parameters such as overall volume, porosity, local thickness and identifying printing defects. The imaging was conducted at the University of Southampton’s μ-VIS X-ray Imaging Centre Centre (https://muvis.org) 3D X-ray Histology facility (https://xrayhistology.org), employing a customized μCT scanner optimized for 3D X-ray histology based on Nikon’s XTH225ST system (Nikon Metrology, UK). Operating at 80 kVp/88 μA (7.4 W), the source-to-object distance was set to 22.7 mm and a source-to-detector distance to 908.4 mm, resulting in a magnification factor of 40x. The acquisition parameters involved collection of 3001 projections, each averaging 4 frames per projection, with an exposure time of 89 ms per projection. The 2850 × 2850 dexels detector was binned 2x (virtual detector: 1425 × 1425 dexels), yielding an isotropic voxel edge of 7.5 μm. Subsequently, the reconstructed data underwent visualization and analysis using Dragonfly software (Comet Technologies Canada Inc.; software accessible at http://www.theobjects.com/dragonfly).^41^ Pore space segmentation was achieved by initially applying a threshold to delineate the “material” and the air followed by manual refinement. The porous space was defined as the difference between the material and a “closed and filled” mask of the material, where “closed and filled” volume mask refers to a new, pores-free object volume derived by morphologically closing the “material” binary mask and filling all inner voids. Porosity percentage was calculated as the ratio between the “closed and filled” volume of the object and the total volume of the pores. Porosity analysis in terms of size distribution was conducted using the connected components tool following a watershed-based separation of the touching pores. Finally, the analysis of the local thickness was conducted using the “Volume Thickness Map” tool, also in Dragonfly, applied to the segmented (binary) object, which comprises the material excluding pores. Local thickness is defined on a per-voxel (3D pixel) basis as the “diameter of the largest sphere that fits inside the object and contains the voxel”. Visual representation of local thickness histograms illustrates the number of voxels associated with a specific sphere diameter (Figure 5).

#### Permeability Modeling

The permeability modeling was conducted in Avizo (Avizo 3D 2023.2), based on the volume images obtained from μCT, and the analysis was focused on a single spike, as porosity characteristics were similar across the volume. The analysis was conducted deploying both a volumetric finite element (FE) model and a pore network model. For the FE-based flow analysis, a central cuboid of the binarized pore space of the target spike was extracted. This cuboid had dimensions of 120 × 90 × 280 voxels, measuring 0.90 × 0.67 × 2.10 mm. From it, any disconnected or isolated pore spaces were removed, as these pores do not directly contribute to permeability. Absolute permeability in both the longitudinal (*Z*) and transverse (*X*) axes was then computed separately. This involved numerically solving Navier–Stokes equations on the binarized pore space.^42,43^ The input-output pressure differential was set to 1 kPa, with the cuboid boundaries parallel to the direction of flow assumed to be impervious. Fluid viscosity was considered as 0.001 Pa·s and a convergence criterion of 1 × 10^–-5^ was applied. The resulting vector velocity field results were used to compute flow tortuosity, defined as the sum of vector magnitudes divided by the sum of components in the flow direction.^44^ Absolute permeability and hydraulic tortuosity were also computed deploying a much simpler Pore Network Model (PNM) approach of the pore space. This involved segmenting the connected pore space of the cuboid subvolume into individual pores using a watershed-based method. The pores and their interpore boundaries (pore throats) were then modeled as spheres and cylinders, respectively. The PNM simulation computed permeability by imposing the same pressure differential of 1 kPa as before across the network. The PNM simulation computes permeability invoking Poiseuille’s law (assuming viscosity = 0.001 Pa·s) and solving for steady flow with no net accumulation within the pores.^43^ Similar to the Navier–Stokes approach, pores were assumed to be fully saturated and boundaries parallel to the direction of flow were considered impermeable. Hydraulic tortuosity was computed by the PNM as the sum of the velocity magnitudes of each pore throat divided by the sum of the velocity magnitude components in the flow direction.

#### Statistical Analysis

All results are presented as mean ± standard deviation of at least three individual experiments. One-way analysis of variance (ANOVA) was used to determine statistical significance set at *p* < 0.05.

## Results and Discussion

### SLS 3D Printing

The printer was prepared as described in the Materials and Methods section (*SLS 3D printing preparation*) and SLS printing was carried out according to the following procedure (Figure 2a). A powder dispenser deposits an adequate amount of powder onto the printing platform. A counter rotating rod (roller) spreads and flattens the powder on the printing bed, creating a consistent powder layer. The platform moves below a laser setup and sintering takes place according to a computer-controlled system which commands laser activation and the printing platform’s movements for the given layer. Then, the printing bed lowers by the set layer height, the platform passes below the powder dispenser and another powder layer is being deposited on top of the sintered one. The previous steps are being repeated for every layer until the final product has been printed. Upon completion of the printing process, the products are collected with a sieve and loose, unsintered powder is removed under mild airflow.

Preliminary material screening studies yielded PVA 4–88 as the preferable binder powder, as the printed prototypes exhibited favorable properties, such as good rigidity (Section S3). Although PVA has been used for SLS printing before, specifically, in the development of naproxen and indomethacin tablets, sintering agents (i.e., active carbon and silica-based pigments) were always used to enhance the laser energy absorption of the powder formulation.^19,22,45^ The incorporation of such a sintering agent may generate concerns regarding safety issues and possible interactions with the API (Section S3).^46^ In our case, however, the C–H bonds that PVA contains, sufficiently absorb the CO_2_ laser’s light (λ = 10.6 μm) and no sintering agents were required.^47^

### Printing Process Optimization

**Figure 3:**
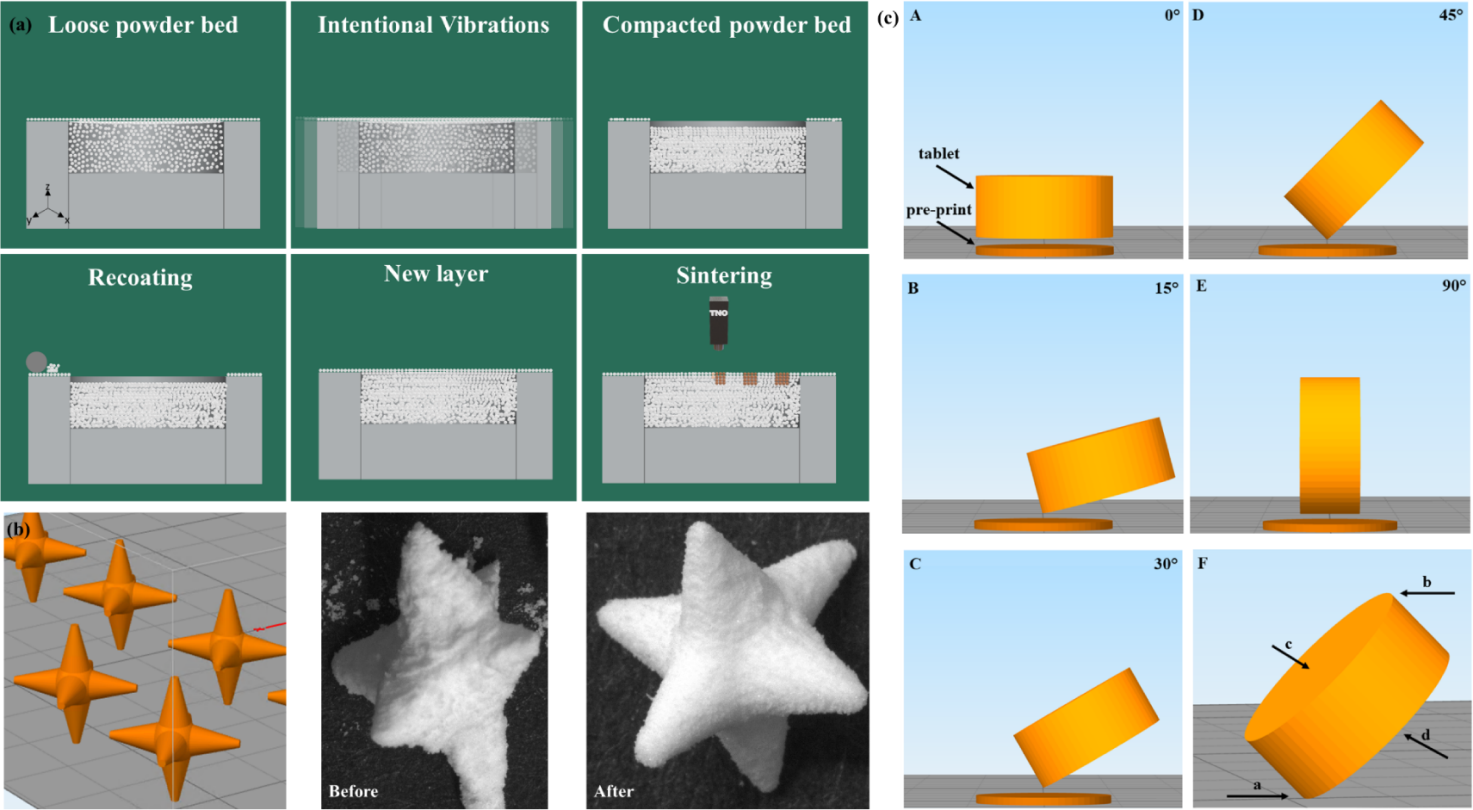
(a) Schematic representation of powder bed compaction through intentional vibrating steps. (b) Printed objects before and after optimization of printing parameters. (c) A–E: the preprint concept and printing at different orientations (0°, 15°, 30°, 45°, 90°) with respect to the printing platform; F: the arrows indicate the different sides and surfaces of the tablet.

High printing and traveling speeds are appealing for time-efficient manufacturing, especially for mass production. High speed printing, however, is not problem-free. In order to counterbalance the effect of the increase of velocity on the energy density, a higher laser power has to be applied (eq S1). In an ambient conditions system, in the absence of a heated printing environment, high laser power means very rapidly occurring local temperature differences on the powder bed surface, which can often lead to material shrinkage and layer warping, as the material cools down. Furthermore, since the printing setup used for this research has a moving printing platform, very rapid changes on X and Y axes, especially during printing of the spiked geometries and their infill, result in an excessively shaking powder bed, layer shifting and eventually, fuzzy products with poor rigidity (Figure 3b). The final optimized printing parameters which have routinely been applied for PVA and the drug-loaded samples are shown in Table S1. The printed prototype of a spiked ball with 6 spikes, after optimization of the traveling and laser parameters, is presented in Figure 2d.

Tablets printed with the aforementioned parameters had a high printing accuracy on the parallel-to-bed plane (*X* and *Y* axes) with an average diameter of 8.97 ± 0.03 mm, corresponding to an average deviation of −0.33% from the digital design (9.00 mm). On the *Z* axis, the tablets have an average height of 4.28 ± 0.09 mm, corresponding to an average deviation of +7.00% from the digital design (4.00 mm). The dimensions of the printed tablets remain consistent among tablets of the same or different batches. It is believed that the height deviation can be attributed to powder adhered on the bottom of the tablets due to a high laser depth focus (higher than one-layer-thickness: 0.10 mm) as well as indirect heating of the powder bed during sintering of the first layer. In addition, during the recoating steps, it is possible that the roller pushes the already printed part deeper into the powder bed and, as a result, the fresh layer that is being deposited on top of the already printed structure is thicker than one-layer-height. The additional powder adherence is also considered responsible for the curved bottom surfaces that often characterize the SLS printed bottom parts that have been designed to be flat. The geometrical issue of increased thickness could be resolved by exploring parametrical offsets in order to meet the required geometry. However, this uncontrolled powder adherence presents an additional hurdle for pharmaceutical manufacturing, mainly because of the difficulty to predict the accurate API-load along with the unpredictable mechanical and dissolution properties of this area of the printed object. Accurate API-loading is of paramount importance, especially in case of prescription of potent drugs and/or the administration to sensitive patient populations, such as the pediatric one.^49^ Moreover, the relatively small units-per-batch number that personalized medicine might require does not allow accomplishment of full per-batch quality-by-testing procedures that large-scale production is currently pursuing. Thus, quality-by-design and product quality assurance is a prerequisite for the implementation of such a technology in pharmaceutical manufacturing.^50^

In order to further evaluate the phenomenon of indirect sintering and the influence of object orientation during printing on their properties, cylindrical tablets were additionally printed at six different angles, namely at 0°, 15°, 30°, 45°, 60° and 90° with respect to the printing bed, as shown in Figure 3c. In the absence of preprints (Figure 3c-F), the side of the tablet that was printed during the first layers (Figure 3c-F) appeared as a curved protrusion on the cylinder, while the ones printed during the last layers appeared flat. The designed-flat surfaces of the tablet (top and bottom of the cylinder) appeared smoother than when printing with a 0° orientation. Overall, the printed objects differed significantly from the mother design with respect to their shape and size. Preprinting discs before the actual tablets did significantly improve the curving issue on the bottom sides.

During a printing job, not the actual printing (activated laser) but the new layer deposition is the most time-consuming process. Hence, a smaller number of layers means faster production times. For a layer height of 0.10 mm, the actual tablet (D: 9.00 mm, H: 4.00 mm) requires 40 layers of printing when printing with a 0° angle and 90 layers when printing with a 90° angle. It is evident that the overall time increases significantly as the orientation angle increases, due to the increase of the layers required.

Recent studies have shown that the mechanical properties of the printed objects depend on the printing orientation, mainly due to different particle sintering within and between the printed layers. Specifically, the hardness of PVA- and PVP/VA-based tablets has been reported to decrease with increasing printing angles. In our case, tablets printed under all orientations were rigid enough to withstand uniaxial, diametral compression loads of 400 N (TesT 112, TesT GmbH, Germany), when applied using a cylindrical probe (D: 8.00 mm).

### Preprint

With the purpose of meeting the aforementioned challenge of additional powder adherence and its unpredictable properties, we explored the concept of printing thin cylindrical discs (so-called “pre-prints”) below the actual tablets (Figure 3c). On top of the preprints, a spacing layer of powder was deposited and the actual tablets were printed on top of it. The discs had a nominal diameter equal to the tablets’ (9.00 mm) and a nominal height of five layers (0.50 mm). Preprints of a larger diameter or height did not further improve printability. The powder spacer between the preprints and the actual tablets had a thickness of two layers (0.20 mm). According to recent studies that conducted in situ thermal analysis of the SLS process, many temperature fluctuations occur during the different printing stages. The maximum temperature is being observed after sintering the infill, upon which deposition of fresh powder takes place, resulting in a sharp drop in temperature.^51^ We suppose that upon deposition of fresh powder on the warm preprints, it adheres on top of them, becoming unavailable for adhering on the bottom surface of the actual tablets. In addition, the preprints offer support against the forces that might push the tablets into the powder bed when applying new layers of powder on top of them, as described in the *Printing Process Optimization* section.

### Vibrations by Design

Many commercially available SLS printers comprise a static printing platform and a scanning laser setup, allowing printing on a relatively motionless powder bed, with some mechanical impacts only during layer application and flattening by a roller or blade. In contrast, our system comprises a moving platform and, as a result, the bed is not static but it receives the impact of various movements that the printing platform is pursuing. An example of this impact has already been described in the *Printing Process Optimization* section where traveling parameters significantly affected the powder bed’s stiffness, as well as the products of the respective printing jobs.

Another issue that has consistently been encountered during this study, is the separation of the printed objects into two rigid pieces, upon collection from the printer, due to poor sintering after a specific number of printed layers. The platform’s rapid movements and sudden changes of direction create vibrations on the powder bed, forcing it to get compacted. This gradual compaction creates a powder bed of decreasing height and volume (i.e., increasing density). Hence, for every new layer, although the printing bed is moving down one-layer-height (0.10 mm), the actual height is larger. After a critical point, a layer thicker than the focused laser voxel is being created causing inadequate sintering with the previous layer.

Taking advantage of the traveling capacities of the moving platform, intentional vibrations were developed in a controlled manner and for each layer to compact the powder bed. Short (0.5 mm) and fast (*v* = 800 mm/s) repeating movements of the printing platform on the *X* and *Y* axes, followed by extra powder deposition and recoating, allow the reproducible and uniform formation of a denser powder bed. Thus, intentional vibrations prevented cumulative deviations and issues with intralayer adhesion, as described above. While compacted powder beds can typically be obtained after repeating tap movements on the *Z* axis, it is supposed that the compaction occurring as a result of movements in the horizontal plane is similar. Intentional vibrating steps were routinely implemented in the .gcode files used for the printing jobs, not only for the powder foundation on which printing will take place, but after every new layer deposition too (Figure 3a).

The effect of the intentional vibrating steps has been assessed with respect to the powder bed density. Hereto, recoating steps were applied until full coverage of the printing bed, which was set in a position that will allow a 2.2 mm thick powder bed to be built. Then, the bed was lowered down, unscrewed and weighed on a digital balance. The percentage mass difference of a powder bed before and after receiving intentional vibration indicated the respective density difference. For PVA, a 7.84% density increase has been measured after intentional vibrations. PVA Parteck MXP 4–88 is a fine powder with a Carr’s index value of 35 ± 1.54, indicating a powder with relatively poor flow, high cohesiveness and high compressibility. It becomes evident that various mechanical impacts during printing can have a significant influence on the density of the powder bed. No layer-shifting was observed during the vibrating steps, the compacted unsintered powder bed was able to sufficiently support the printed structures.

As previous studies have demonstrated, printing speed, and therefore energy density (eq S1), can have a high impact on products’ porosity, and thus significantly affect their dissolution behavior, with higher energy densities producing denser products with a slower drug release.^18,52,53^ Although low thermal energy absorption may facilitate printing through sintering of the powder particles, the higher amount of thermal energy absorbed by the powder bed might cause softening or melting of the polymer. As the temperature increases, the amorphous polymer softens and flows to fill the voids between the neighboring solid particles creating cavities on top of the printed layer which, upon deposition of fresh powder, are filled with additional powder. If not adequately filled, the cavities can cause variations in sintering among layers of the same tablet.^19,53^ The implemented vibrations by design, however, offer the opportunity for a predictable and constant powder bed density as well as for products with higher density. SLS products are typically characterized by high porosity. For pharmaceutical applications and in combination with the appropriate polymeric materials, SLS therefore enables the creation of rapidly dissolving dosage forms.^54,55^ However, high porosity means low solid content and possibly a low API content. This can reduce the flexibility of dose adjustment, or it might necessitate the administration of more than one units-per-dose in order to meet the requirements of the therapeutic regimen.^49^ A powder bed of higher density enables higher drug-loadings in the same tablet volume. In addition, intentional powder compaction secures homogeneity and consistency along the printed geometry. Mechanical compaction in line with the other printing stages capacitates printing of tablets of gradual or high density, without the hazard of material degradation due to exposure to high thermal energy. Moreover, it introduces a new set of parametrical configurations, subjected to tunable variations, and paves the way for attributes of controlled-release.

### Characterization of the 3D Printed Objects

The optimized 3D printing protocol comprises the operating parameters as shown in Table S1, in addition to an object orientation of 0° in respect with the printing bed, and the use of thin preprints below every actual object (Figure 3c). Intentional vibrations were applied on the powder foundation as well as after every new layer deposition (Figure 3). Placebo PVA tablets (*N* = 20) printed with the aforementioned method had an average diameter of 8.97 ± 0.03 mm and an average height of 4.07 ± 0.09 mm. Before applying the developed protocol, the respective values were diameter: 8.97 ± 0.03 mm and height: 4.28 ± 0.07 mm. The nominal values were 9.00 mm and 4.00 mm, respectively. A 4 times reduction of deviation from the design (from +7.00% to +1.75%) on the *Z* axis was achieved by means of the incorporation of preprints and intentional vibrations. No object separation was reported (as described in the *Vibrations by design* section) and the printing process remained very precise on the *X*–*Y* plane. Similarly, the LOP-loaded tablets had an average diameter of 8.94 ± 0.03 mm and an average height of 4.04 ± 0.03 mm. The LOP-loaded tablets had an average mass of 134.3 ± 3.0 mg and they were characterized by low average weight variability (2.23%) within the same and between different batches. According to the European Pharmacopoeia version 5.0 (2.9.5. Uniformity of mass of single-dose preparations) a maximum deviation of 7.5% is allowed for tablets with an average mass between 80 and less than 250 mg. Furthermore, the individual mass of no more than 2 of the 20 units may deviate from the average mass by a percentage greater than 10%, but the mass of no unit may deviate by more than 15%. In our experiments no printed tablets had a mass deviation of more than 3.32% from the average mass, meaning that the printing process is suitable for producing pharmaceutical tablets with low weight variability. The recorded friability of the printed tablets was 0.88%, and thus in compliance with the guidelines of the European Pharmacopoeia version 10.0 (2.9.7. Friability of uncoated tablets) which requires a friability of <1 wt % for uncoated tablets. More complex designs (e.g., a 6-spiked ball) were printed with high dimensional accuracy as well. The distance between the tip surfaces of two parallel spikes was assessed using a “ray-tracing thickness” algorithm (Figure 2e). The results indicated a distance of 8.09 mm when printed on the horizontal plane and 7.86 mm when printed on the Z axis, exhibiting a −1.82% and a −4.6% deviation from the nominal value (8.24 mm).

### Characterization of the 3D Printed Spiked Balls by Means of Microfocus Computed Tomography (μCT)

In order to assess the internal structural response to mechanical loading and the failure of the spiky protrusions, one of the spikes was manually snapped off before imaging. The aim was to qualitatively assess the potential introduction of any significant voids or defects that might emerge during the failure process. This could provide insights into the broader resilience of the printed specimens.

Total volumes of the pore-free (volume-filled) object were measured 33.4 mm^3^ and 39.9 mm^3^ for the 5-spikes and 6-spikes object, respectively. Porosity analysis showed that both structures had similar total porosity, amounting to 43.8% and 44.3%, respectively suggesting that porosity is primarily influenced by the printing method and the properties of the raw material rather than being determined by the structure (Figure S2).

**Figure 4:**
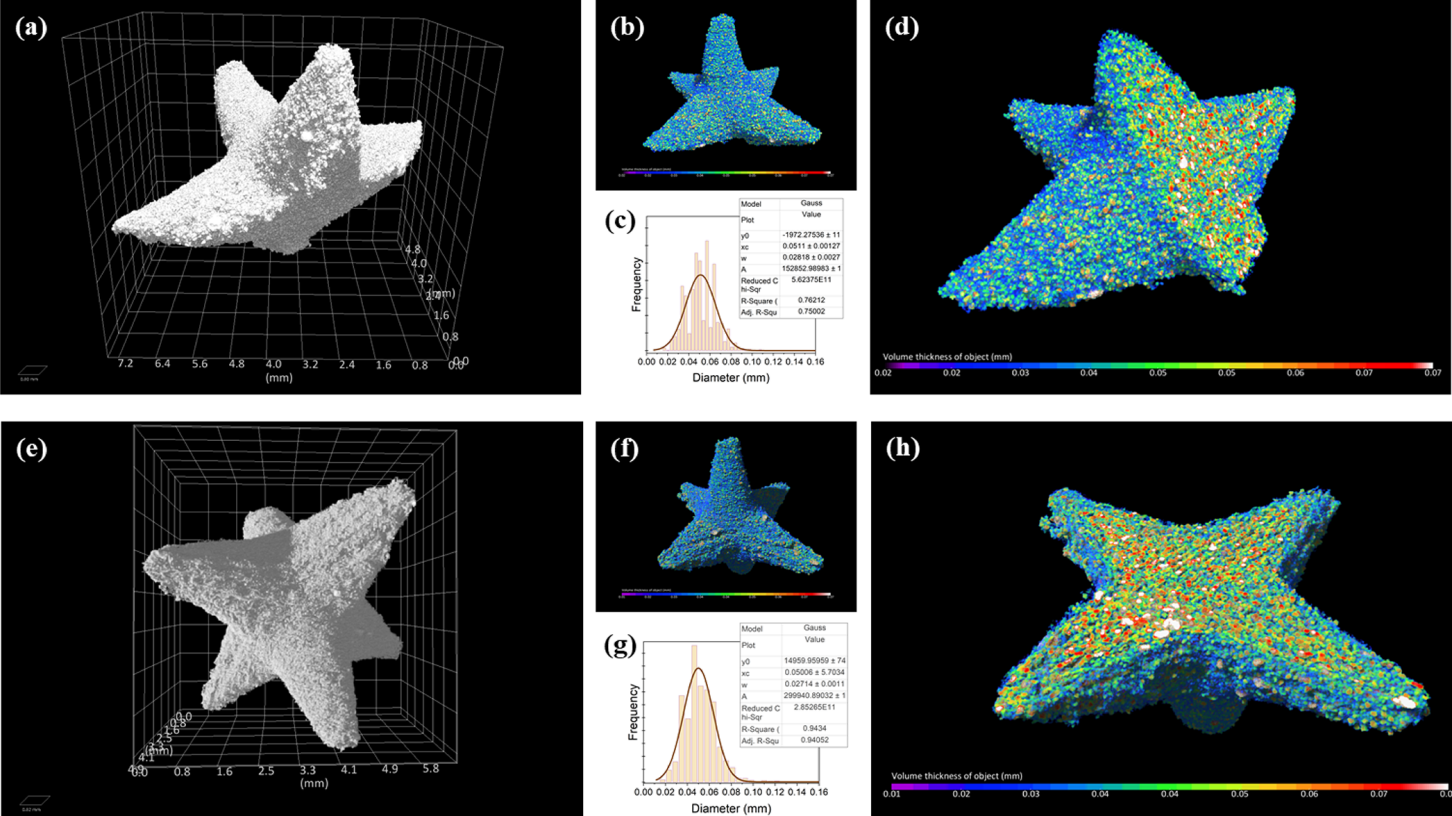
Spiked balls with 5-spikes (top) and 6-spikes (bottom): (A and E) 3D photorealistic representations of the printed objects; (B and F) 3D volume rendering displaying the local thickness; (C and G) “clipped” volume rendering illustrating the thickness in the object’s core. Core-color scales range: up [0.02–0.07], down [0.01–0.07]; (D and H) histogram depicting the distribution of local thickness values.

Local thickness visualization of the fused matrix of the two objects is presented in Figure 4 (color-scale bar from 0 to 0.07 mm). Local thickness follows normal distribution, with the peak center at 0.05 mm, and a full width at half-maximum (fwhm) of 0.03 mm. This suggests a relatively uniform thickness of approximately 50 ± 30 μm across the fused elements.

**Figure 5:**
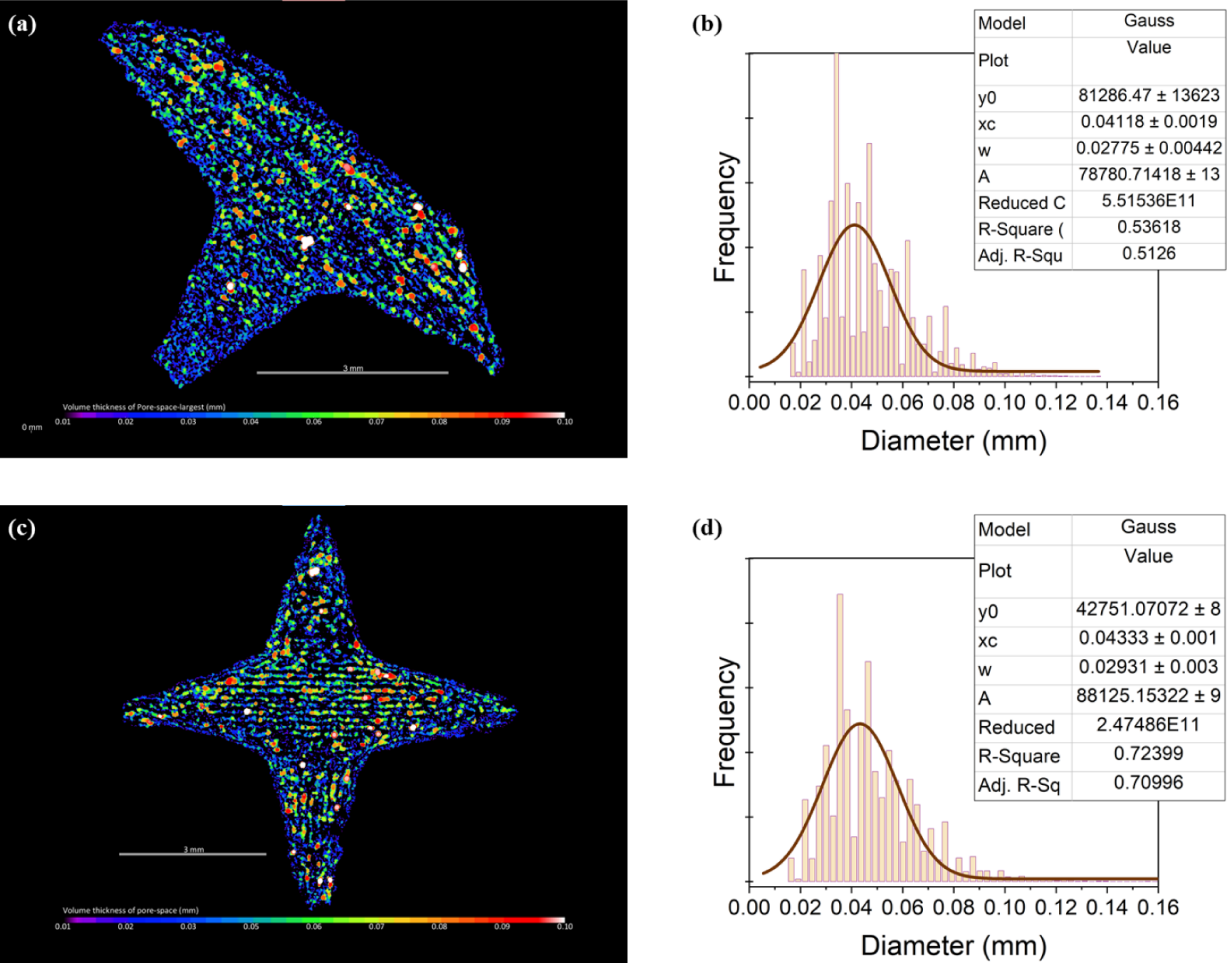
Thickness distribution of the pore phase in both the 5-spikes (top) and 6-spikes (bottom) objects, along with their respective local thickness maps; scale bar at 3 mm, color bar from 0.01 mm to 0.10 mm.

Thickness analysis applied to the pore space showed identical trends. It is worth noting though, that the majority of the pores were interconnected making it difficult to define “individual” pores for analyzing single-pores’ characteristics. For this reason a thickness-analysis was selected as a more appropriate approach to characterize the pores-phase. Analysis of thickness unveiled a porous network characterized by local thickness distributions with peak center at 0.04 mm and a fwhm of 0.03 mm. Similarly to the matrix phase, these results also indicate a consistent pore-size distribution, with an equivalent diameter of approximately 40 ± 30 μm (Figure 5).

The strong interconnectivity mentioned above resulted in the watershed-based segmentation of the pores domain returned an oversegmented image. While analysis of these results as single-pores’ properties might not be an accurate representation of single-pores’ properties, they can still provide useful information about pore clusters, which resemble larger equivalent pores. The connected component analysis results are shown in Table S4 and indicate pore-cluster volumes of approximately 0.0015 mm^3^ with an aspect ratio of these clusters of 0.44–0.45.

Interestingly, if we use this aspect ratio information and assume that the pores are ellipsoidal with a minor axis equal to the *mean local thickness* (0.04 mm), we can calculate the pore volume using eq 1) based on this local thickness and aspect ratio. For pores where the *Local Thickness mean* equals *a* = *b*/2 = *c*/2 (flattened ellipsoid pores), the calculated pore volume is 0.0010 mm^3^. For pores with a shape where the local thickness mean equals *a* = *b* = *c*/2 (more spherical pores), the volume is 0.0005 mm^3^. These estimated volumes align closely with those obtained from connected component analysis.
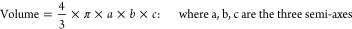
1

### Solid State Analysis

**Figure 6:**
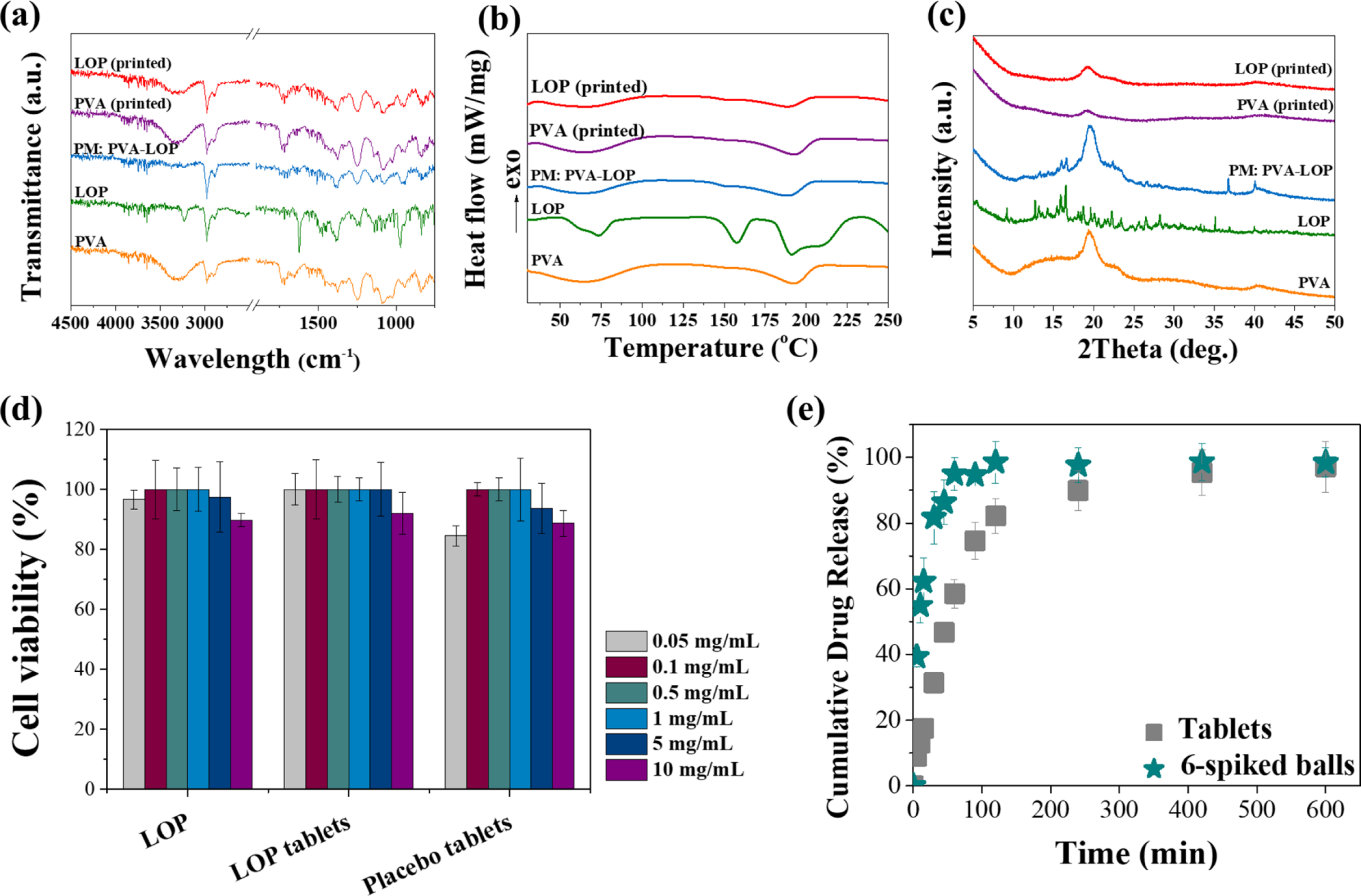
(a) FTIR spectra; (b) DSC spectra; (c) X-ray diffractograms; (d) cell viability; (e) in vitro drug release profiles from 3D printed tablets and 6-spiked balls.

Solid state analysis was performed to examine possible interactions between the components in the formulation as well as the influence of the overall printing procedure (Figure 6). The FTIR spectrum of pristine PVA shows a broad band between 3200 and 3550 cm^–1^, which refers to O–H stretching from the intermolecular and intramolecular hydrogen bonds (Figure 6a). The band between 2840 and 3020 cm^–1^ corresponds to the C–H stretching from alkyl groups. The C=O stretching from the acetate group appeared at 1734 cm^–1^, which is attributed to residual admixtures in the PVA.^56,57^ The bands at 1417–1457 cm^–1^ and the bands at 1085–1190 cm^–1^ are assigned to −CH_2_ and C–O–C bonds, respectively. The spectrum of pristine LOP shows characteristic peaks at 3223 cm^–1^ and at 2855–3020 cm^–1^, corresponding to hydrogen bonded O–H and the C–H bonds of the alkyl chain, respectively. The C=O bond of the amide group appears at 1626 cm^–1^ and the C–C bonds of the aromatic ring appear at 1447 cm^–1^. The peaks at 1375 cm^–1^ and at 1385 cm^–1^ refer to the −CH_3_ bonds.^58−60^ In the PVA-LOP physical mixture, the FTIR spectrum shows shifts suggesting the potential molecular interactions between the two components. The broad O–H stretching of PVA shifts 3300 cm^–1^ to 3260 cm^–1^, indicating a reduction in free hydroxyl groups and the formation of hydrogen bonds with LOP (Figure S4). The O–H stretching vibration of LOP shifts from 3223 to 3365 cm^–1^, further supporting the presence of intermolecular hydrogen bonding. The printing process appears to alter the physical state of the components, as evidenced by the FTIR spectrum of the printed formulations (LOP-printed). The O–H stretching band remains shifted compared to the pristine components, confirming that hydrogen bonding between PVA and LOP persists after printing (Figure S4).

DSC analysis confirms the results above (Figure 6b). The thermogram of pure LOP exhibited an endothermal peak at 190.8 °C, indicative of a melting event corresponding to its tetrahydrated form.^58^ Pure PVA powder exhibited a broad melting endotherm at *T*_peak_ = 192.5 °C. The thermogram of the binary mixture of PVA with LOP exhibits characteristic endothermal events for both compounds, confirming that the solid state of the materials was not modified during regular blending. In contrast, signs of crystalline LOP are no longer visible in the LOP-loaded printed tablets.

Diffractograms of placebo and LOP-loaded tablets, physical powder blend and raw materials are presented in Figure 6c. The strong peak corresponding to PVA at 2θ = 19.33° is consistent in all diffractograms. Numerous distinctive peaks of pure LOP were seen in its diffraction pattern, representative of its crystalline state. In the physical blend with the excipient, prominent peaks (2θ = 13.31°, 15.99°, 16.57°, 22.30°, 26.64°) were visible at approximately the same 2θ positions with raw LOP but with reduced intensities, probably due to dilution.^61^ XRD analysis reveals partial amorphization of LOP within the PVA matrix after printing while the physical mixture shows partial crystallinity of LOP within the PVA matrix. The selective laser sintering process enhances these effects, disrupting the crystalline structure of LOP and promoting a more homogeneous distribution of the drug within the polymeric matrix.

Although, the physical blend of PVA with LOP exhibits characteristic properties for both materials, no peaks corresponding to the drug could be detected in the thermogram, X-ray diffractogram or FTIR spectrum of the LOP-loaded printed tablets, indicating possible molecular dispersion of the API in the printed objects. In addition, physicochemical comparison of pristine PVA powder and recycled powder (Figure S3) (i.e., used in at least 10 printing jobs) showed no significant physicochemical differences indicating the possibility of reusing the unsintered powder in future printing jobs without significant alterations on product quality (Section S4).

### Biocompatibility

The biocompatibility assessment indicates that Caco-2 cells proliferate well when exposed to pure LOP, placebo and LOP-loaded 3D printed objects (Figure 6d). After 24 h of culture, all groups exhibited cell viability of at least 80%, indicating no cytotoxicity of all specimens. A slide decrease on cell viability could be observed in high concentrations (5–10 μg/mL) of the studied materials which can be attributed to a possible decrease in the pH of the medium, combined with an increase in the viscosity of the medium, due to water absorption by PVA, that is believed to affect cell proliferation.

### In Vitro Drug Release

Each dosage form had a drug content close to the theoretical one (8.00 wt %). Specifically, drug loading was calculated to be 7.83% (±0.23%) and 7.60% (±0.32%) for the LOP-loaded 3D printed tablets and 6-spiked balls, respectively. The deviations from the theoretical value might be attributed to less efficient binding of the API on the surface of the dosage forms, provoking its loss during handling.

*In vitro* release of LOP from 3D printed tablets and 6-spiked balls was evaluated in PBS (pH 7.4) (Figure 6e). A total drug release was observed for the tablet (cylindrical shape) after 10 h, with more than 80% been released within the first 2 h. A faster drug release was observed for the 6-spiked balls, achieving total drug release within 2 h. It becomes evident that the geometry of the dosage form significantly influences the rate of drug dissolution. Upon immersion in the dissolution medium, the PVA matrix initially forms a hydrocolloid structure which swells and then gradually dissolves. As it has been reported before, the dose and the size of PVA-based dosage forms do not influence the relative drug release, whereas the surface area to absolute volume ratio (SA/V) significantly does.^62,63^ The 6-spiked balls had an SA/V ratio of 36.08 mm^–1^ while the respective average value for the printed tablets was remarkably lower, equal to 1.48 mm^–1^. A smaller SA/V ratio means longer diffusion pathways and consequently, a smaller drug dissolution rate.

### Permeability Modeling

Permeability (*k*) represents the resistance to flow within a porous medium and is significantly influenced by the structure of the pore network, including factors such as pore size, pore size distribution, interconnectivity, and tortuosity. These characteristics affect the internal surface area per unit length, and in turn viscous drag and the resistance to flow; for example higher degree of interconnected and tortuous pores increase the viscous drag and the resistance to flow.^64^ Permeability modeling provides an invaluable tool for better understanding the dosage form–medium interactions that underpin dissolution mechanisms, and drug release behavior of these formulations.

Along the longitudinal axis, absolute FE-based permeability measured 28.97 d (2.86 × 10^–11^ m^2^), while in the transverse direction it was 34.06 d (3.36 × 10^–11^m^2^). The FE-based flow tortuosity values were calculated to be 1.344 along the longitudinal axis and 1.295 along the transverse axis. Unlike the FE model, which treat the porous material as a uniform medium, PNM treats it as a network of interconnected pores and the constrictions between these pores are represented as throats. This makes them particularly computationally efficient and as a result the method can be applied to larger and/or more complex pore geometries.

**Figure 7:**
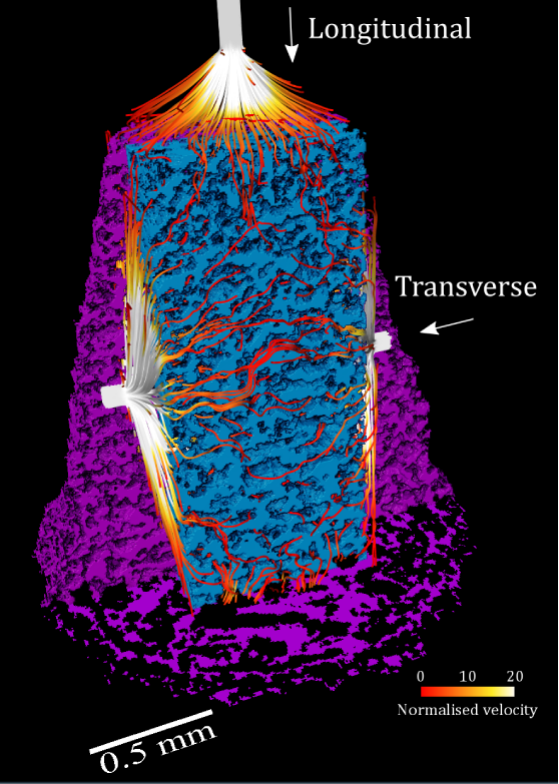
3D representation of flow velocity streamlines resulting from the FE-based permeability analysis, in both longitudinal and transverse directions, normalized by 1,000 μm/s; target spike solids shown in purple, analysis cuboid solids rendered in blue.

The PNM results returned slightly higher tortuosity values compared with the FE method, measuring 1.645 along the longitudinal axis and 1.516 along the transverse axis. In terms of absolute permeability, a slightly higher value was observed along the longitudinal direction, measuring 30.45 d (3.00 × 10^–11^ m^2^). However, the permeability along the transverse axis was nearly double that of the longitudinal direction, registering at 60.62 d (5.98 × 10^–11^ m^2^). This difference may be attributed to the shorter flow path and reduced diversity in pore and throat sizes, potentially resulting in an oversimplified network configuration in the PNM model along this direction. It is important to note that Navier–Stokes simulation results are based on a larger vector field than the PNM results. The former includes data from streamlines located outside the pore space, with directions that diverge widely from the flow direction, due to the point-like nature of the flow origin. These values can be put in context by being compared to the permeability of other typical porous materials. For example, sandstone typically has a permeability ranging from 0.1 to 1000 d depending on several factors including total porosity, pore geometry and grain shape^65,66^ whereas clay typically demonstrates a much lower permeability, ranging from 0.0001 to 1 d.^67^ The measured tortuosity indicates a convoluted propagation path through the interconnected pores and channels along the two axes (tortuosity value of 1 indicates a straight line-path). Tortuosity along the transverse direction was calculated to be slightly lower than longitudinal, which partially explains the elevated value of permeability along that direction. A higher tortuosity along the longitudinal direction indicates a more convoluted flow path, which results in increased flow resistance and slower fluid movement through the porous network in this direction. This could imply quicker dissolution and higher API release rates along transverse direction (cf. Supplementary video) (Figure 7). This direction-dependent differences in fluid flow can be attributed to the anisotropic pore geometry and structure within the printed object, which in turn leads to differences in permeability and tortuosity. We can only speculate at this stage that such differences may be the result of the “printing” direction of the dosage form during fabrication. During SLS printing, certain layers are formed against gravitational forces, potentially impacting the consolidation and pore shape in the vertical (with respect to printing) direction. Meanwhile, surface tension between neighboring particles likely facilitates sintering to “spread” preferentially along the plane parallel to the printing table, creating a varying pore network in the transverse (with respect to printing) direction. This anisotropic sintering behavior may be a key factor contributing to the observed directional differences.

The observed higher permeability and lower tortuosity in the transverse direction suggest faster fluid flow and dissolution rates along this axis direction (cf. Supplementary video) (Figure 7), which could influence the dissolution profile of the dosage form. Intentional or unintentional nonuniformly loaded formulations, where the API is distributed unevenly, such anisotropy in flow properties may lead to characteristic or inconsistent release kinetics, potentially affecting the dosage form’s performance. The interplay between SLS-induced anisotropy and API distribution might be a critical design factor for optimizing the printing parameters. Our findings suggest the potential for directional influences on dissolution and release behavior. However, experimental validation is crucial to verify, validate and quantify these effects, as well as for determining their specific impact on the performance of the dosage form.

### In Vitro Disintegration Time Test and Mucoadhesion Studies on Ex Vivo Intestinal Model

**Figure 8:**
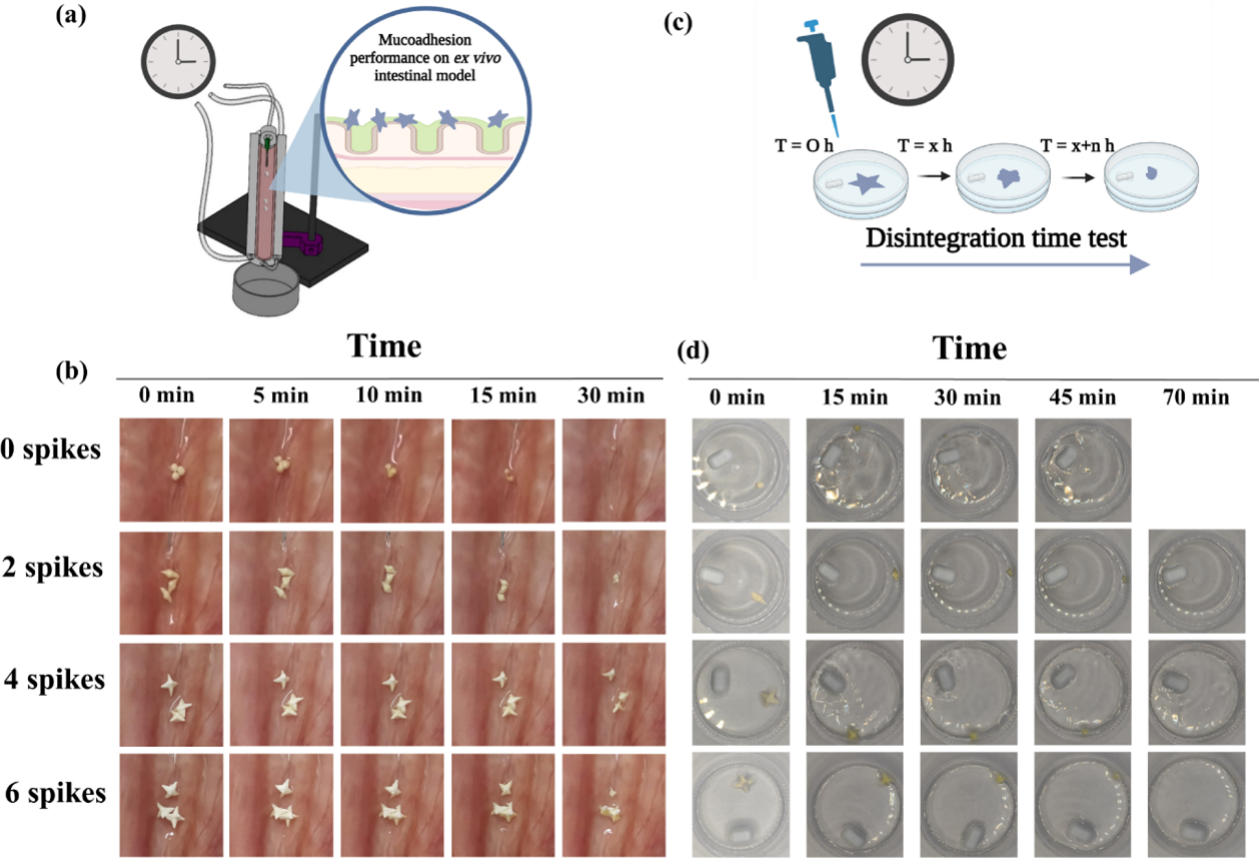
(a) Mucoadhesion performance of the unspiked and spiked balls on an ex vivo intestinal model. (b) Photos capturing the retention time of the unspiked and spiked balls on porcine intestinal tissue during ex vivo performance. (c) Illustration of the setup for determining the disintegration time of the sharp-edged objects. (d) In vitro disintegration test: Photos capturing the balls (without spikes), 2-spiked balls, 4-spiked balls, and 6-spiked balls over time during disintegration test.

The performance of the spiked specimens, and the influence of the number of spikes was evaluated through an *ex vivo* study, as described in the *Ex vivo intestinal model* section (Figure 8a). The behavior of the spiked balls on the intestinal tissue is an indicator of their retention time. The number of spikes had been hypothesized to affect the travel path of the spiked balls on the tissue and hence, their retention time. Specifically, it was expected that the higher the number of spikes, the longer the retention time would be, due to the increasing number of contact points with the tissue. Upon placement on the model, all designs (0, 2, 4, or 6 spikes) immediately adhered on the surface of the tissue. The 2-spiked balls were oriented on the tissue with the spikes parallel to its surface whereas the 4-spiked and the 6-spiked balls retained multiple contact points with it (Figure 8b). No tissue-embedded spikes were observed. No movement was recorded for the spiked designs before their complete dissolution. Consequently, no tissue penetration could be reported. After 16 min, the 0-spiked balls moved as a result of the fluid stream and rolled toward a lower part of the tissue where they remained until complete dissolution. The 0-spiked balls were smaller in size, and mass compared to the spiked configurations, which could have contributed to their shorter retention time. Additionally, the 0-spiked balls began to move after a certain period, indicating that retention time is influenced not only by mass and volume but also by shape. The spiked configurations demonstrated enhanced retention capabilities, likely due to their increased contact points with the tissue, supporting the hypothesis that spiked designs provide improved retention compared to simple, rounded shapes.

We propose the following explanation for this difference. PVA is a nonionic polymer with good mucoadhesive properties, which slowly swells when in contact with aqueous solutions. As swelling progresses, the adhesive properties gradually decrease and a gel-like structure is obtained.^68^ At the same time, as dissolution proceeds, the mass of the solid object decreases. Hence, the resistance to the fluid stream becomes weaker because of its gradually smaller size and decreasing adhesion efficiency. As a result, the unspiked balls were carried away by the fluid stream and relocated. The spiked designs, however, exhibited enhanced adhesion and increased contact time with the mucus layer. The spiked balls remained on the same position, regardless of the size of the undissolved fragments of the printed object. It is important to note that the *ex vivo* model employed in this study does not simulate intestinal peristalsis. While this limitation does not affect the primary objective of evaluating retention time and mucoadhesion, future studies could incorporate peristaltic motion to assess its potential influence on hydrodynamics and retention behavior.

Spiked balls with a bigger number of spikes exhibited longer retention times. These differences can be attributed to, not only differences in the mass of each design, but also deviations among the fractions that were in contact with the fluid stream. Specifically, for spiked balls with a higher number of spikes, a smaller volume fraction was submerged in the PBS medium, hence resulting in a slower dissolution and longer retention time. In contrast, *in vitro* disintegration studies (Figure 8c), where all objects were fully submerged in the medium, showed no significant differences among the disintegration times of spiked balls with different numbers of spikes. An average time of 64.94 min was recorded for their complete *in vitro* disintegration of the spiked balls (63.66 ± 2.08 min, 65.33 ± 1.52 min and 65.83 ± 0.76 min for the 2-spiked, 4-spiked and 6-spiked balls, respectively), while the unspiked balls disintegrated in an average time of 33.0 ± 1.0 min (Figure 8d). While the *ex vi*vo model used in this study provides valuable insights into the adhesive and retention capabilities of these geometries, it does not fully replicate the dynamic environment of the gastrointestinal tract. *In vivo* studies in a large animal model will replicate normal conditions such as peristalsis, mucus turnover, and fluid dynamics, which may influence the performance of these spiked designs and confirm their retention in the gastrointestinal tract.

## Conclusions

The current study reports on the capabilities of SLS 3D printing for the fabrication of drug-loaded specimens for colonic drug-delivery. A promising printing protocol has been proposed for drug product manufacturing with controlled attributes, without the incorporation of sintering agents. With the purpose of creating consistent products, the fabrication protocol comprised printing thin discs below the actual products. Intentional mechanical vibrations were implemented during the printing job in order to achieve uniformity and rigidity of the printed objects, which met pharmacopoeial standards. Sharp-edged geometries of the drug-loaded objects had been hypothesized to impart desirable adhesion properties on the colonic tissue and therefore, increased retention time. The behavior of PVA-based spiked balls was studied on a custom ex vivo intestinal setup, and demonstrated superior properties to the unspiked balls, with respect to adhesion on the tissue. The influence of the number of the spikes was also assessed. Adoption of SLS 3D printing as the manufacturing process enabled flexibility in architectural adjustments and control over tunable properties of the drug-loaded products, such as retention time in the colon.

The versatility of this method lies in its potential for personalization. By modifying the number, shape and arrangement of spikes, the proposed structures can be tailored to optimize retention times or target specific regions within the intestinal tract. For instance, intricate geometries, such as multilobed or asymmetric shapes, could be used to selectively bind to irregular mucosal surfaces, such as those found in diseased tissues (i.e., Crohn’s disease) while minimizing off-target adhesion.^69^ These modifications enable precision delivery improving both efficacy and safety in personalized therapeutic regimens.

## Data Availability

The μCT data and the image-based modeling data for this study is accessible and can be found on Zenodo.org with the following DOI: 10.5281/zenodo.13490759. Researchers interested in accessing the data can use this DOI to locate and download the relevant information. The rest of the data can be made available upon request.

## Supporting Information Available

Permeability video (MP4)
Figure S1: schematic representation of the setup used for the *ex vivo* experiments, Figure S2: image processing steps for the porosity analysis), Figure S3: DSC and FTIR data or pristine and recycled materials, Table S1: optimized printing parameters and the calculated energy density used for printing experiments, Table S2: pharmaceutical materials that were tested for their printability with SLS, Table S3: Carr’s flowability classification of powders based on angle of repose and Carr’s index values, Table S4: connected component analysis results for the 5- and 6- spiked balls (PDF)
