# Supplementary material for: Spiked Systems for Colonic Drug Delivery: Architectural Opportunities and Quality Assurance of Selective Laser Sintering
Source: ACS Biomater Sci Eng. 2025 Feb 6;11(3):1818–33. doi: 10.1021/acsbiomaterials.4c02038 (PMC11897947; doi:10.1021/acsbiomaterials.4c02038)
Supplement: Supplementary file 2 — ab4c02038_si_002.pdf [file ab4c02038_si_002.pdf]

Supplementary information

Spiked systems for colonic drug delivery: architectural opportunities and quality assurance of selective laser sintering

Angelos Gkaragkounis [a,b,], Konstantina Chachlioutaki [a,c], Orestis L. Katsamenis [d,e], Fernando Alvarez-Borges [d], Savvas Koltsakidis [f], Ioannis Partheniadis [a], Nikolaos Bouropoulos [g,h], Ioannis S. Vizirianakis [i], Dimitrios Tzetzis [f], Ioannis Nikolakakis [a], Chris H. J. Verhoeven [b], Dimitrios G. Fatouros [a,c], Kjeld J. C. van Bommel [b,*]*

[a]*Laboratory of Pharmaceutical Technology, Department of Pharmacy, School of Health Sciences, Aristotle University of Thessaloniki, Thessaloniki GR 54124, Greece*

[b]*The Netherlands Organization for Applied Scientific Research (TNO), Eindhoven 5656 AE, The Netherlands*

[c]*Center for Interdisciplinary Research and Innovation (CIRI-AUTH), Thessaloniki, Greece*

[d]*μ-VIS X-Ray Imaging Centre, Faculty of Engineering and Physical Sciences, University of Southampton, Southampton, SO17 1BJ, UK*

[e]*Institute for Life Sciences, University of Southampton, University Road, Highfield, Southampton SO17 1BJ, UK*

[f]*Digital Manufacturing and Materials Characterization Laboratory, School of Science and Technology, International Hellenic University, Thessaloniki, 57001, Greece*

[g]*Department of Materials Science, University of Patras, Patras, 26504 Rio, Greece*

[h]*Foundation for Research and Technology Hellas, Institute of Chemical Engineering and High Temperature Chemical Processes, 26504, Patras, Greece*

[i]*Laboratory of Pharmacology, Department of Pharmacy, Aristotle University of Thessaloniki, Thessaloniki, GR 54124, Greece*

Keywords: selective laser sintering, 3D printing, spiked drug delivery systems, colonic drug delivery, mucoadhesion, loperamide, extended retention time

Material and methods:

S1. Experimental assembly of the ex vivo intestinal model

A custom system was devised and utilized for the *ex vivo* experiments (Figure S1a).This system featured a 3D-printed tissue holder with adjustable angle, measuring 19.50 cm in length and 3.30 cm in width, as previously reported.[1,2] The holder was fixed to a conventional laboratory stand via a 3D-printed bracket and comprised a base for a fluid flush system as well as internal channels facilitating the passage of tubes, responsible for maintaining the holder at a temperature of 37 °C. The 3D-printed parts were designed in Solidworks 2023 (Dassault Systems, USA) and then sliced with Ultimaker Cura 4.2 (the Netherlands). Glycol-modified polyethylene terephthalate (PETG) filament was used for their fabrication on an FDM printer (Creality Ender S1 Pro, Creality, China) employing a 0.4 mm nozzle, a layer height of 0.2 mm and a printing speed of 50 mm/s. The experimental assembly comprised a dynamic vapor sorption (DVS) unit consisting of a humidity generator (GenRH-A, Surface Measurement Systems, UK) and a probe (Rotronic Hygroclip 2 humidity, ±0.8% accuracy), which provided control over relative humidity (RH) in the range of 0–98%. The DVS was equipped with RH and temperature displays and included the following components: an air inlet port fitted with a pressure gauge and relief valve, a flowmeter, a water reservoir for humidifying the air, and a valve for mixing humidified air with dry environmental air to achieve the desired RH (a schematic representation of the DVS unit can be found elsewhere). [3] Additionally, the DVS was connected via a USB interface to a computer installed with appropriate software, which controlled the RH and duration of air supply and received signals from the probe. The RH recording over the experimental period is presented in **Figure S1c**. It is evident that RH was maintained mainly around 60%, confirming the adequacy of control. Downward spikes

represent the beginning of each trial, during which the door of the cabinet was opened to introduce the specimens to the tissue. The temperature of the PETG tissue holder and the colonic tissue was measured using a thermal imager (Uni-T UTI260B, Uni-Trend Technology (China) Co., Ltd., China) with a 256*192 thermal resolution **(Figure S1b)**. The emissivity value (ε) of 0.92 was used for PETG, and for the tissue, the emissivity value of pig skin (i.e., 0.98) was employed. [4,5]

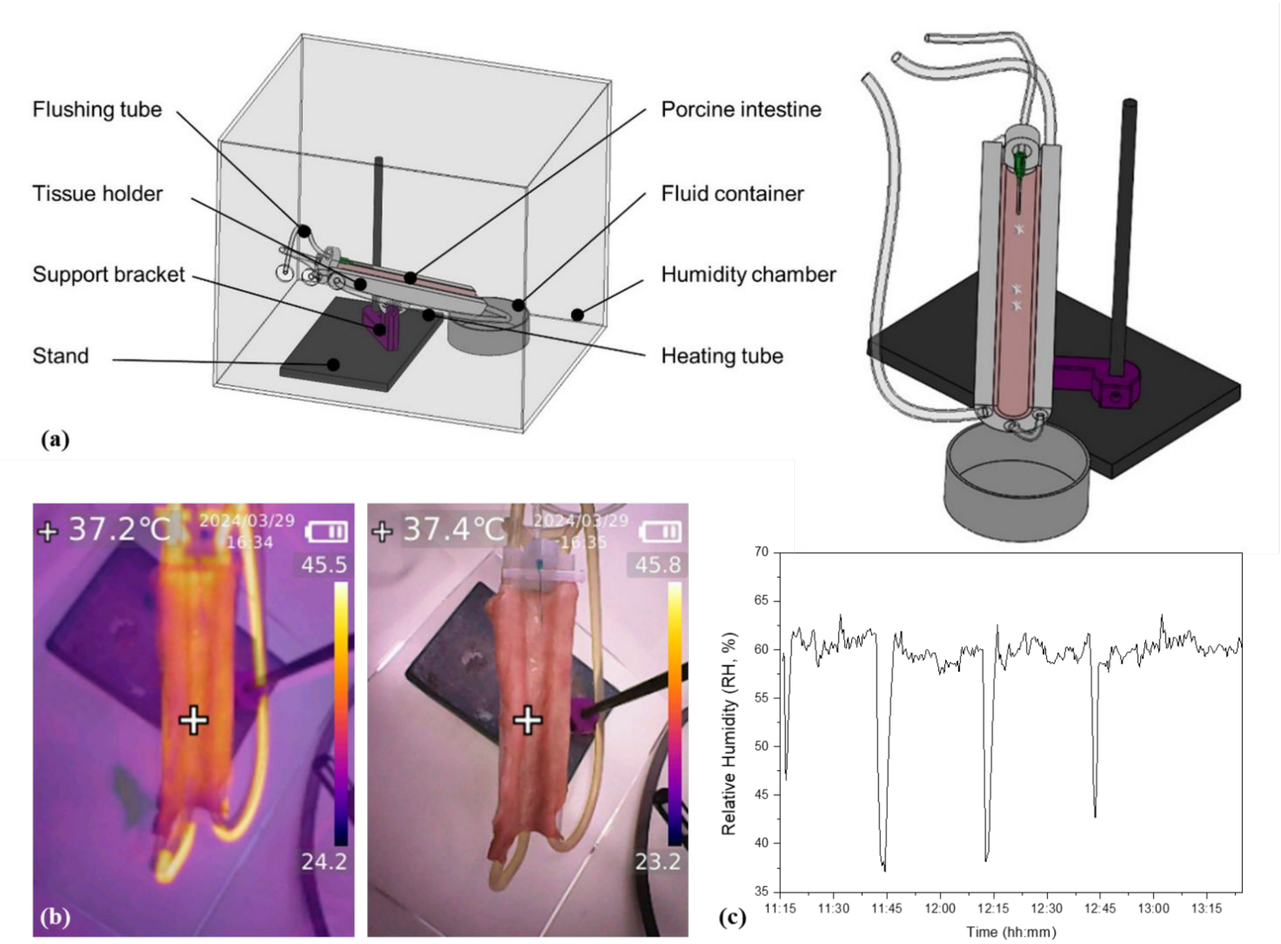

Figure S1. (a) Schematic representation of the setup used for the *ex vivo* experiments; **(b)** Thermal imaging of the intestinal tissue while on secured on the tissue holder; **(c)** Relative humidity recording during the *ex vivo* experiments.

Results:

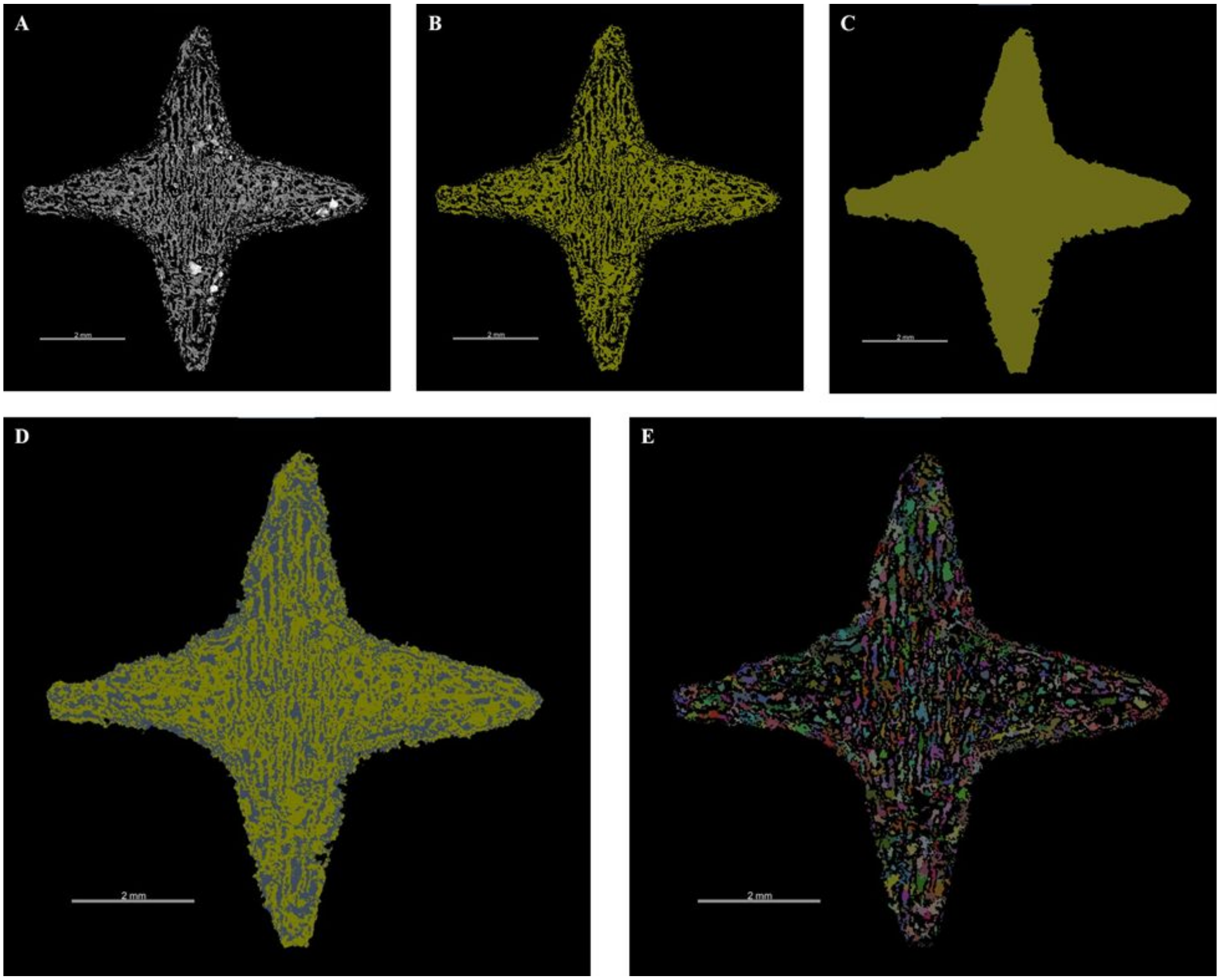

Figure S2. Image processing steps for the porosity analysis: (A) representative grayscale slice from the µCT volume; (B) threshold-based segmentation of the material followed by (C) the "closed and filled" mask encapsulating the entire object, including its porous space. (D) Subtracting B from C results into the porous space which is shown in (D) in blue, providing a visual contrast between the two phases. The porous population after applying the watershed separation algorithm is shown in (E), where each individualized pore is rendered in a different color.

S3. Material screening

The first part of this work focused on exploring multiple pharmaceutical materials for their potential to meet the objective of this study: SLS printing of spiked geometries. The particle fusion that takes place during the SLS process is a result of heat energy transfer among them. The optical properties of the powder determine the quantity of the initial light energy that will be transformed into thermal energy and thus cause sintering. Until now, common pharmaceutical polymers have been proven to be poor absorbers of light in the UV-Vis and IR ranges that many commercially available SLS printers operate on. Consequently, a major consideration for the successful sintering of pharmaceuticals, has been the incorporation of a sintering agent (i.e. a compound that will enhance laser beam absorption). The current sintering agents, with Candurin Gold Sheen (a mixture of potassium aluminum silicate, iron oxide and titanium dioxide) being the most common one, have been used in quantities that already reach safety limits. Hence, it was an ambition of this project to identify materials and formulations that would be printable with the current instrumentation without the addition of a sintering agent. [6,7]

A trial-and-error approach was applied in which at least 15 common pharmaceutical materials (Table S1) were tested in combination with a broad range of energy densities. The energy density of the primary laser beam is the cumulative parameter that defines the properties of the printed structures. If all parameters are constant, the energy density (ρ_E) may be calculated by the following Equation (S1):

$$\rho_E = \frac{P}{v * HS * LH} \tag{S1}$$

where P is the laser power (mW), v is the printing speed (mm/s), HS is the hatching distance (mm) and LH is the layer height (mm).

Two grades of PVA (4-88 and 3-82) and one grade of HPMC were proven to be well printable. Their printed products were rigid enough and could withstand mild handling. The spiked object were characterized by high geometrical accuracy, in comparison to their digital mother-designs.

A large proportion of particles being smaller than 100 μm in the initial powder, and the ability to form smooth layers during printing were essential considerations for the selection of an appropriate matrix material in order to achieve fine architectural characteristics, like the ones regarding the spiked geometries of the present study. The maximum powder particle size determines the minimum layer height that can be employed in the printing process and affects product smoothness and printing resolution. A favorable rigidity of the resulting printed products and absence of serious discoloration during printing yielded PVA 4-88 as the preferable binder powder to focus on in this study. The outcome of three screening mixtures of PVA 4-88 (PVA 4-88: HPMC 50:50 wt.%, PVA 4-88:lactose 50:50 wt.%, PVA 4-88:PEG 8K 50:50 wt.%) was formulations with acceptable printability and printed product with good rigidity, proving that PVA 4-88 can act as a binder in complex formulations, including those investigated here. Not only that, material combinations may mean combinations of properties which create opportunities for achieving enhanced mechanical and chemical characteristics, such as controlled release.

S4. Recycling of powder material

Heated powder pools and heaters of the upper active layer are often being used during SLS printing to bring the temperature of the powder bed close to the glass-transition (T_g) or melting temperature

of the binder material. In that case, usually only minor laser energy absorption is required to trigger fusion. Furthermore, a controlled-temperature system compensates for temperature differences that might occur during the different printing stages. [10,11] As a consequence, all the powder used in this process has a thermal history, which may be detrimental, as it can lead to degradation of many pharmaceutical materials, especially in the case of sensitive APIs. Prolonged thermal exposure of semi-crystalline polymers can have several effects: a) a change in the degree and/or type of crystallinity, b) a change of molecular weight, c) other intra- or intermolecular reactions and d) discoloration. [12] These effects may transform the material into a state unsuitable for further use or modify the printed products' properties when recycling the materials. During our study, no additional heat had been applied, except of the heat generated on the powder by the laser beam. Thus, unsintered powder had a minimal thermal history, with any heat load mainly being due to indirect heat transfer. Physicochemical comparison of pristine PVA 4-88 powder and recycled powder (i.e. used in at least 10 printing jobs) indicated no significant physicochemical differences. All major peaks related to PVA 4-88 were observed in the FTIR spectrum of the recycled powder which, according to DSC analysis, exhibits a similar thermal profile with the pristine powder (Figure S3). Typically, the unsintered powder is being mixed with virgin powder and reused. For pharmaceutical purposes, validity and repeatability of such a method (which should be unique for every combination of materials and printing conditions) has yet to be examined for its compliance with Pharmacopoeias' guidelines. Nevertheless, the creation of any unsintered powder that is inappropriate for recycling raises the financial and environmental costs. Recently, selective powder deposition on predetermined places on the printing bed has been applied for the reduction of the powder volumes. [13]

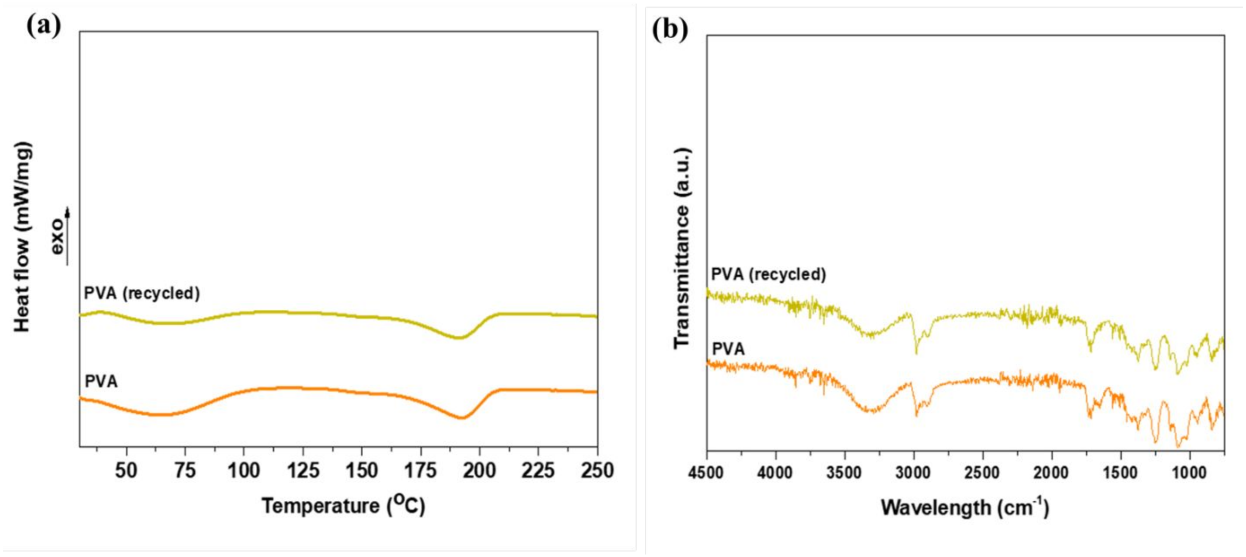

Figure S3. (a) DSC thermograms and (b) FTIR spectra of pristine and recycled PVA powders

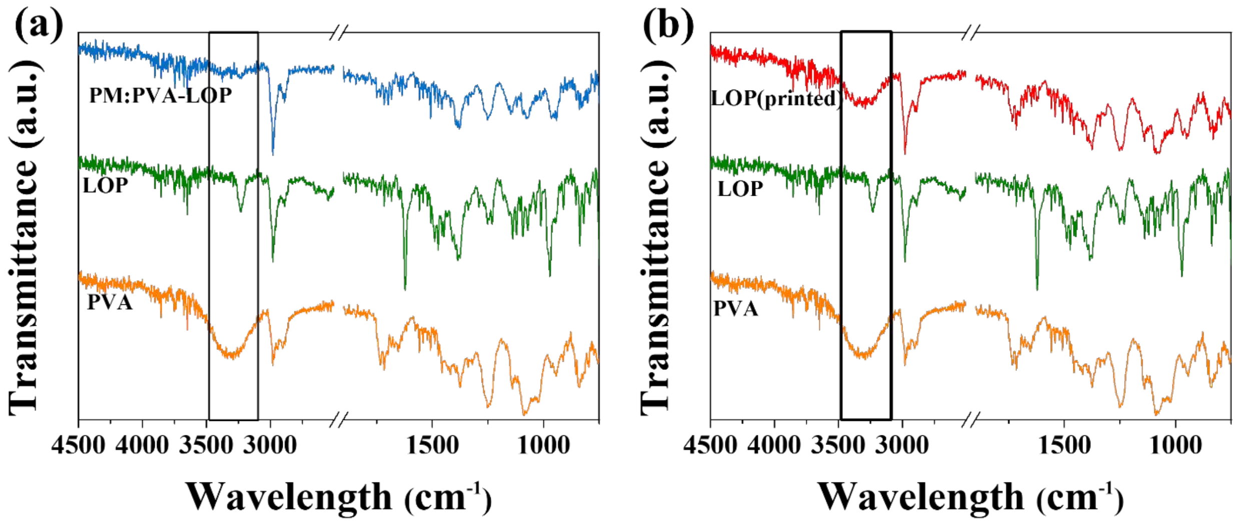

Figure S4. FTIR spectra of pristine PVA, LOP, physical mixture of PVA-LOP and 3D printed LOP.

Table S1. Optimized printing parameters and the calculated energy density used for printing experiments with PVA 4-88.

Parameter	Optimal Value	Tested range
Laser Power (nominal)	166 mW	8.58 – 1216 mW
Layer height	0.10 mm	-
Hatching distance	0.15 mm	0.10 - 0.50 mm
Printing speed	20 mm/s	10 – 20 mm/s
Travelling speed (*X*, *Y* axes)	83 mm/s	15 – 100 mm/s
Acceleration (*X, Y* axes)	50 mm/s^2	10 – 50 mm/s^2
Jerk (*X, Y* axes)	50 mm/s	10 – 50 mm/s^2
Infill percentage	100 %	90-100 %
Infill pattern	Rectilinear (0°, 45°, 90°, 135°)	-
Thickness of starting powder bed	2.2 mm	-
Perimeter scans	1	0-4
Calculated energy density	533.3 mJ/mm^3	85.8 – 4053.3 mJ/mm^3
**Vibrating steps**		
Travelling distance	0.50 mm	0.50-1.00 mm
Travelling speed (*X*, *Y* axes)	800 mm/s	500 – 800 mm/s

Table S2. Pharmaceutical materials that were tested for their printability with SLS and comments regarding their properties, after visual inspection.

Material	Brand	Printability	Rigidity	Discoloration	Comments
PVA 4-88	Parteck® MXP	Very high	Very high	Low	Fraction of PSD < 106 µm: Very high
PVA 3-82	Parteck® MXP	Very high	Very high	Low	Fraction of PSD < 106 µm: High
HPMC	Sigma Aldrich	Very high	Very high	Very high	Fraction of PSD < 106 µm: Low
PVA 05	Vivapharm®	High	High	Low	Fraction of PSD < 106 µm: Fair
HPMC E50	Vivapharm®	High	High	High	Fraction of PSD < 106 µm: Low

HPMC E6	Vivapharm®	Fair	High	High	Fraction of PSD < 106 µm: Low
Vinylpyrrolidone-vinyl acetate	Kollidon® VA64	Fair	Very low	Low	Fraction of PSD < 106 µm: Fair
Polycaprolactone (PCL)	Sigma Aldrich	Very Low	Low	Low	Fraction of PSD < 106 µm: Very low
PVP/VA 64	Vivapharm®	N/A	N/A	N/A	Could not collect from the printing bed
Microcrystaline Cellulose	Pharmacel® 102	N/A	N/A	Low	Could not collect from the printing bed
Methacrylic acid- Methyl Methacrylate copolymer	Eudragit® L100	N/A	N/A	N/A	Could not collect from the printing bed

Table S1. Carr's flowability classification of powders based on angle of repose [Flowability (AoR)] and Carr's index [Flowability (CI)].

Material	Angle of Repose (°)	Flowability (AoR)	Carr’s index	Flowability (CI)
PVA 4-88	41.42 ± 0.59	Passable	35 ± 1.54	Poor
PVA 3-82	41.96 ± 0.84	Passable	27.66 ± 0.67	Poor
HPMC	32.87 ± 0.97	Good	20.35 ± 2.33	Passable

Table S2. Connected component analysis results for the 5- and 6- spiked balls.

	**5-spiked object**		**6-spikes object**	
	Average ± SD	Median	Average ± SD	Median
Volume (mm^3)	0.0017 ± 0.0015	0.0013	0.0018 ± 0.0015	0.0013
Aspect Ratio	0.45 ± 0.14	0.4479	0.44 ± 0.14	0.42

References

(1) Vaut, L.; Scarano, E.; Tosello, G.; Boisen, A. Fully Replicable and Automated Retention Measurement Setup for Characterization of Bio-Adhesion. *HardwareX* **2019**, *6*, e00071. https://doi.org/10.1016/j.ohx.2019.e00071.

(2) Dalskov Mosgaard, M.; Strindberg, S.; Abid, Z.; Singh Petersen, R.; Højlund Eklund Thamdrup, L.; Joukainen Andersen, A.; Sylvest Keller, S.; Müllertz, A.; Hagner Nielsen, L.; Boisen, A. Ex Vivo Intestinal Perfusion Model for Investigating Mucoadhesion of Microcontainers. *International Journal of Pharmaceutics* **2019**, *570*, 118658. https://doi.org/10.1016/j.ijpharm.2019.118658.

(3) Partheniadis, I.; Kopanelou, D.; Gamlen, M.; Nikolakakis, I. Monitoring the Weight and Dimensional Expansion of Pyridostigmine Bromide Tablets under Dynamic Vapor Sorption and Impact of Deliquescence on Tablet Strength and Drug Release. *International Journal of Pharmaceutics* **2021**, *609*, 121150. https://doi.org/10.1016/j.ijpharm.2021.121150.

(4) Badarinath, R.; Prabhu, V. Real-Time Sensing of Output Polymer Flow Temperature and Volumetric Flowrate in Fused Filament Fabrication Process. *Materials* **2022**, *15* (2), 618. https://doi.org/10.3390/ma15020618.

(5) Soerensen, D. D.; Clausen, S.; Mercer, J. B.; Pedersen, L. J. Determining the Emissivity of Pig Skin for Accurate Infrared Thermography. *Computers and Electronics in Agriculture* **2014**, *109*, 52–58. https://doi.org/10.1016/j.compag.2014.09.003.

(6) Zhang, Y.; Thakkar, R.; Zhang, J.; Lu, A.; Duggal, I.; Pillai, A.; Wang, J.; Aghda, N. H.; Maniruzzaman, M. Investigating the Use of Magnetic Nanoparticles As Alternative Sintering Agents in Selective Laser Sintering (SLS) 3D Printing of Oral Tablets. *ACS Biomater. Sci. Eng.* **2023**. https://doi.org/10.1021/acsbiomaterials.2c00299.

(7) Gueche, Y. A.; Sanchez-Ballester, N. M.; Bataille, B.; Aubert, A.; Leclercq, L.; Rossi, J.-C.; Soulairol, I. Selective Laser Sintering of Solid Oral Dosage Forms with Copovidone and Paracetamol Using a CO2 Laser. *Pharmaceutics* **2021**, *13* (2), 160. https://doi.org/10.3390/pharmaceutics13020160.

(8) Tikhomirov, E.; Ahlén, M.; Di Gallo, N.; Strømme, M.; Kipping, T.; Quodbach, J.; Lindh, J. Selective Laser Sintering Additive Manufacturing of Dosage Forms: Effect of Powder Formulation and Process Parameters on the Physical Properties of Printed Tablets. *International Journal of Pharmaceutics* **2023**, 122780. https://doi.org/10.1016/j.ijpharm.2023.122780.

(9) Török, J.; Kaščak, J.; Kočiško, M.; Telišková, M.; Dobránsky, J. Orientation of the Model in SLS Printing and Its Influence on Mechanical Properities. *7* (4), 8.

(10) Tikhomirov, E.; Åhlén, M.; Strømme, M.; Lindh, J. In Situ Thermal Image Analysis of Selective Laser Sintering for Oral Dosage Form Manufacturing. *Journal of Pharmaceutical and Biomedical Analysis* **2023**, 115396. https://doi.org/10.1016/j.jpba.2023.115396.

(11) Gueche, Y. A.; Sanchez-Ballester, N. M.; Bataille, B.; Aubert, A.; Rossi, J.-C.; Soulairol, I. A QbD Approach for Evaluating the Effect of Selective Laser Sintering Parameters on Printability and Properties of Solid Oral Forms. *Pharmaceutics* **2021**, *13* (10), 1701. https://doi.org/10.3390/pharmaceutics13101701.

(12) Bashir, Z.; Gu, H.; Yang, L. Evaluation of Poly(Ethylene Terephthalate) Powder as a Material for Selective Laser Sintering, and Characterization of Printed Part. *Polymer Engineering & Science* **2018**, *58* (10), 1888–1900. https://doi.org/10.1002/pen.24797.

(13) Kopp, S.-P.; Medvedev, V.; Tangermann-Gerk, K.; Wöltinger, N.; Rothfelder, R.; Graßl, F.; Heinrich, M. R.; Januskaite, P.; Goyanes, A.; Basit, A. W.; Roth, S.; Schmidt, M. Electrophotographic 3D Printing of Pharmaceutical Films. *Additive Manufacturing* **2023**, *73*, 103707. https://doi.org/10.1016/j.addma.2023.103707.
